# Laccase Properties, Physiological Functions, and Evolution

**DOI:** 10.3390/ijms21030966

**Published:** 2020-01-31

**Authors:** Grzegorz Janusz, Anna Pawlik, Urszula Świderska-Burek, Jolanta Polak, Justyna Sulej, Anna Jarosz-Wilkołazka, Andrzej Paszczyński

**Affiliations:** 1 Department of Biochemistry and Biotechnology, Maria Curie-Skłodowska University, Akademicka 19 Street, 20-033 Lublin, Poland; anna.pawlik@poczta.umcs.lublin.pl (A.P.); jpolak@poczta.umcs.lublin.pl (J.P.); justyna.sulej@poczta.umcs.lublin.pl (J.S.); anna.wilkolazka@poczta.umcs.lublin.pl (A.J.-W.); 2 Department of Botany, Mycology and Ecology, Maria Curie-Skłodowska University, Akademicka 19 Street, 20-033 Lublin, Poland; urszula.swiderska-burek@poczta.umcs.lublin.pl; 3 Professor Emeritus, School of Food Science, University of Idaho, Moscow, ID 83844, USA; andrzej@uidaho.edu

**Keywords:** laccase, function, multicopper oxidase, polyphenol oxidase, lignin, melanin, evolution

## Abstract

Discovered in 1883, laccase is one of the first enzymes ever described. Now, after almost 140 years of research, it seems that this copper-containing protein with a number of unique catalytic properties is widely distributed across all kingdoms of life. Laccase belongs to the superfamily of multicopper oxidases (MCOs)—a group of enzymes comprising many proteins with different substrate specificities and diverse biological functions. The presence of cupredoxin-like domains allows all MCOs to reduce oxygen to water without producing harmful byproducts. This review describes structural characteristics and plausible evolution of laccase in different taxonomic groups. The remarkable catalytic abilities and broad substrate specificity of laccases are described in relation to other copper-containing MCOs. Through an exhaustive analysis of laccase roles in different taxa, we find that this enzyme evolved to serve an important, common, and protective function in living systems.

## 1. Introduction

As the oxygen concentration in the biosphere increases due to the action of cyanobacteria, which started releasing photosynthetic oxygen to the Earth’s atmosphere about 2.45 billion years ago, the widely available and water soluble FeII (ferrous ion) gradually oxidizes to FeIII (ferric ion) with practically no solubility in water and very limited availability to the living systems. In response to these changes, aerobic microbes developed highly sophisticated iron uptake (siderophores) and management systems [1], and the appearance of atmospheric oxygen created evolutionary pressure to find bioavailable metal(s) with iron-like high redox potentials. Therefore, the importance of copper (CuII/CuI) and manganese (MnIII/MnII) increased, especially in aerobic organisms as they embraced a similar but not identical role for iron (FeIII/FeII). Among many biological functions, copper interacts with a wide range of proteins to drive diverse structures and, hence, different biochemical reactions [1].

In general, copper-containing proteins, almost all extracellular, are widely distributed in nature, where they participate in oxygen transport and activation and electron(s) transfer in redox processes [2]. Multicopper oxidases belonging to this group are capable of oxidation of a wide range of substrates using oxygen as an electron acceptor. Multicopper oxidases (MCOs) reduce O_2_ to H_2_O without releasing harmful, partially reduced O_2_ molecules called reactive oxygen species (ROS). MCOs encompass laccases (EC 1.10.3.2) and a large family of copper oxidases, which include, among others, ascorbate oxidases (EC 1.10.3.3), ceruloplasmin (EC 1.16.3.1), bilirubin oxidase (EC 1.3.3.5), phenoxazinone synthase (EC 1.10.3.4), and metallo-oxidase Fet3p (EC 1.16.3.1). The amino acid sequences of all multicopper oxidases contain a small, 10–20 kDa, cupredoxin-like domain and possess relatively simple 3D structures, primarily composed of beta sheets and turns. They mainly serve as electron transfer proteins [3,4]. In recent years laccase structure was analyzed many times by means of crystallography. However, most of the described proteins are of a fungal or bacterial origin. Perhaps low amounts and methodically complicated purification protocols for plant and animal laccases make them difficult to be obtained as crystals. Nevertheless, it is proven that despite their wide taxonomic distribution and diversity of substrates, the molecular architecture of laccases is common for all multicopper oxidases [5,6].

**Figure 1:**
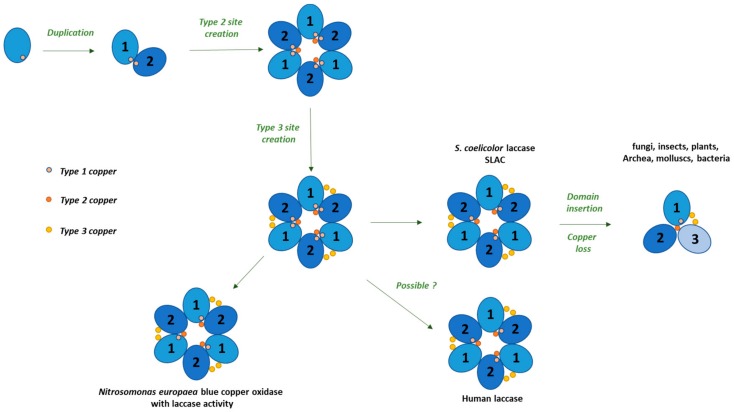
Schematic representation of the molecular evolution of laccases (based on Komori and Higuchi [11] and Nakamura and Go [16]). The oval shapes represent blue-copper-binding sites. The classes of protein domains are marked in pale and dark blue. Dots represent copper types.

**Figure 2:**
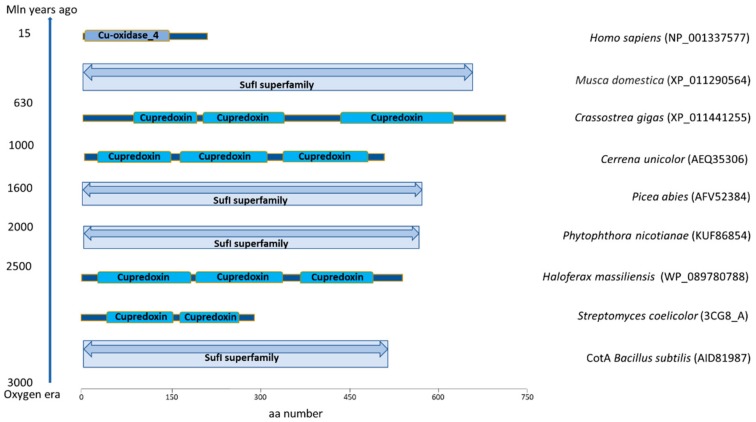
Conserved cupredoxin (cyan) laccase domains in different taxonomic groups. Cu-oxidase_4 is a multicopper polyphenol oxidoreductase laccase. Suf I represents a multicopper oxidase with three cupredoxin domains (includes cell division protein FtsP and spore coat protein CotA). The conserved domains were retrieved from the NCBI database. The appearance of organisms is shown in the timeline on the left in milliom (mln) years.

The evolutionary tree of MCOs suggests a complex process in which different proteins, including taxonomically diverse laccases, most likely originated (Figure 1). In contrast to lignolytic peroxidases, which in the timeline of evolution appeared just before fungi gained the ability to decompose lignin [7], laccases are most likely one of the earliest metalloproteins, as they were present in organisms at the early evolutionary stages. Therefore, their lignolytic function is rather a result of substrate specialization and most likely the secondary function of these most frequently studied enzymes in the group of MCOs, which are widely distributed in fungi and bacteria and occur in higher plants and animals as well [8]. Based on the number of cupredoxin-like domains, MCOs are classified as two-domain (2dMCO), three-domain (3dMCO), and six-domain (6dMCO) enzymes [9]. Laccases usually contain three domains, like those described in fungi, plants, insects, and some bacteria, mostly composed of about 500 aa. In the past decade, small bacterial laccases (about 200 aa) were discovered and classified as 2dMCOs [3], and it was suggested that three-domain enzymes evolved from two-domain bacterial proteins (Figure 1 and Figure 2). However, it is hard to elucidate the origin of short sequences (c.a. 170 aa) from vertebrates, which were annotated in recent years and some of them are biochemically categorized as laccases [10]. There is a chance that they may form a separate branch of MCO evolution, as their structures are rather close to prokaryotic laccases (Figure 1 and Figure 2). Moreover, some fungal species, for example *Pleurotus ostreatus*, also produce laccases that are smaller than the typical 3dMCO and consist of only two domains [11]. It is highly probable that, during evolution, bacterial 2dMCO appeared before fungal 3dMCO (Figure 1 and Figure 2). Despite the low amino acid sequence homology between fungal and bacterial laccases, their molecular architecture is similar, and the overall geometry of their active sites is highly conserved [5,12,13]. Three dimensional structure predictions for bacterial laccase suggest three sequentially arranged cupredoxin-like domains [5,13] and copper ligands arranged in four conserved motifs (HXHG, HXH, HXXHXH, and HCHXXXHXXXXM/L/F) typical for the MCO family [14,15].

Therefore, questions arise whether small fungal laccases are a missing link between bacterial 2dMCO and fungal 3dMCO or whether fungi evolved 2dMCO independently by losing one domain. A similar process may have occurred in the animal kingdom. However, only fungi are known to produce 2dMCO and 3dMCO enzymes. Most laccases in a given species are coded by several genes, which is not an unusual phenomenon especially in eukaryotes. In most cases, this is caused by the need for large amounts of the gene product or catalytic subfunctionalization of a particular isozyme. A number of recent papers described the differential expression of laccases as a response to diverse environmental factors [17,18], which most likely require synthesis of laccases with different substrate specificity and kinetic properties [19,20]. When considering numerous copies of laccase genes in many organisms and their diverse functions, subfunctionalization seems more convincing [21].

**Figure 3:**
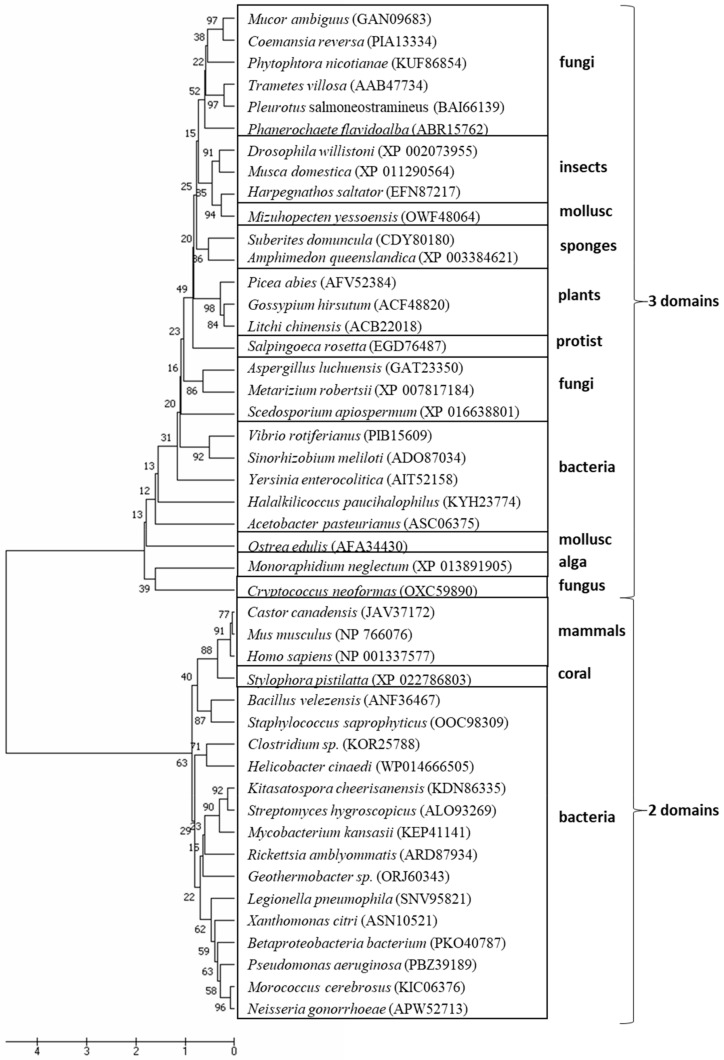
The evolutionary history of laccase was created using the UPGMA method [22]. The optimal tree with the sum of branch length = 38.42825656 is shown. The percentage of replicate trees in which the associated taxa clustered together in the bootstrap test (1000 replicates) is shown next to the branches [23]. The tree is drawn to scale, with branch lengths in the same units as those of the evolutionary distances used to infer the phylogenetic tree. The evolutionary distances were computed using the Dayhoff matrix based method [24] and expressed in the units of the number of amino acid substitutions per site. The analysis involved 46 amino acid sequences. All positions containing gaps and missing data were eliminated. In total, there are 72 positions in the final dataset. The evolutionary analyses were conducted in MEGA7 [25].

Interestingly, the human *LACC1* gene shows no similarity to the other mammalian proteins, since it contains a C-terminal domain homologous to bacterial multicopper polyphenol oxidases (PO) and laccase [10]. Alignment of laccase sequences across the tree of life shows that they are grouped into main clades: two- and three-domain oxidases (Figure 3). The two-domain bacterial, mammalian, and coral laccases are clustered together in one clade. Not all laccases are two-domain; some species, including fungi, plants, and insects have three-domain laccases. The organisms clustered in these two separate groups comprise taxonomically different species (Figure 1). It should also be noted that two- and three-domain of these enzymes are present in animals and bacteria (Figure 2) and some Ascomycota laccases are more like those in plants and bacteria, which in turn are closer to insects than fungal enzymes. Moreover, *Cryptococcus* (animal and plant fungal pathogen) and *Monoraphidium* (green algae) laccases are grouped together separately from plant and fungal enzymes. It seems that this enzyme evolved from a single ancestral protein and later differentiated into structurally and functionally novel laccases across the tree of life; this process is expected to continue in the future.

## 2. Laccase as a Versatile Biocatalyst

The biologically important attributes of laccases include the broad substrate spectrum and the use of molecular oxygen as a final electron acceptor. The initial electron acceptor in laccase-catalyzed oxidation is copper T1 located in the cavity close to the enzyme surface [26]. The reduction of copper T1 is a rate-limiting step in the reactions catalyzed by laccase and the relatively low values of the T1 redox potential (from 420 to 790 mV vs. normal hydrogen electrode (NHE)), which limit the laccase substrate array to molecules containing phenolic moieties [27]. Phenolic compounds are oxidized by laccase to phenoxyl radicals, which then engage in either coupling-based polymerization or radical rearrangement. However, depending on phenoxyl radical stability, redox reversibility featuring oxidation of a targeted substrate is observed. Radical-based coupling or redox recycling of phenolic substrates acting as mediators broadens the spectrum of laccase substrates [28].

Depending on the redox potential, laccases are divided into two groups: low- and high-redox-potential enzymes. Low-redox-potential enzymes occur in bacteria, plants, and insects, whereas high-redox-potential laccases are widely distributed in fungi [29]. Laccases catalyze both anabolic and catabolic reactions. Representative catabolic processes include degradation of lignin and humus by fungal laccases. Laccase-catalyzed anabolic reactions are involved in morphogenesis; for example, they catalyze polymeric pigment synthesis [30], cuticle sclerotization [31], polyflavonoid synthesis [32], lignification [33], and humidification of soil organic matter [34]. In anabolic processes, the redox potential of plant, bacterial, and insect laccases makes radical coupling reactions thermodynamically possible without additional chemicals [35]. Typical substrates for these reactions are low molecular weight phenolic compounds yielding several types of polymeric products such as pigments, lignins, polyflavonoids, and humus [32,36]. Purified laccases from plant tissues utilize monolignols and flavonoids as substrates for radical-based polymerization of lignin and polyflavonoid synthesis, as in the case of *Eucalyptus* lignin [37] or seed coat formation in *Arabidopsis thaliana* [38]. Oligomeric products derived from such reactions are randomly covalently coupled into cell wall components of plant fibers, like in *Populus trichocarpa* [39]. Similar radical-based coupling is described for laccases bounded to the insect cuticle matrix [40]. Cuticle sclerotization via laccase-mediated protein cross-linking processes was observed in the case of *Manduca sexta* and *Tribolium castaneum* [41]. Typical substrates in the cuticle sclerotization are catechol, *N*-acetyldopamine, and *N*-β-alanyldopamine, which are transformed by laccase into quinones, followed by radical coupling of histidyl residues of cuticular proteins [42]. The cross-linking of these low molecular weight substrates to protein-based matrices is also essential for cuticle pigmentation, hardening, water-resistance, and protection against environmental stresses [31]. The main pigments synthesized by laccase are melanins formed from endogenous substrates such as dihydroxynaphthalene [43,44] and exogenous substrates such as homogentisic acid and dihydroxyphenylalanine [45]. Synthesis of the melanin pigment through laccase-catalyzed polymerization of the precursors mentioned above is described in *C. neoformans* [45] and *Aspergillus fumigatus* [44].

Catabolic processes performed by laccases require the generation of free radicals, which then oxidize target compounds such as lignin and humus. The first group of radicals is usually produced by fungal laccases from natural methoxyhydroquinones. Next, these radicals initiate Fenton reaction leading to production of different ROS [46,47]. The second group of radicals is generated by oxidation of natural low molecular weight redox mediators often derived from oxidized target substrates. For example, preferential oxidation of phenolic lignin units leads to the release of small phenolic residues with oxidized side chains [28]. In addition to the phenolic hydroxyl group, these compounds may contain additional functional residues, such as methoxyl, amine, ketone, aldehyde, or carboxyl. Such mediator-based oxidations occur principally in basidiomycetes, which are very efficient lignocellulose decomposers. Laccases alone do not depolymerize native lignin, but rather modify its surface [48]. However, the exact attack mechanisms and the enzymatic features of laccases in relation to lignin modification are presently unclear because of the lack of methods [29] to analyze these interactions. In the case of model phenolic compounds of lignin, laccase cleaves the bonds between C_1_ and C_2_ carbons, known as C_α_–C_β_ cleavage, and between C_α_ and the aryl group, which is known as alkyl–aryl cleavage, without the use of a mediator [49]. It is suggested that laccase can cleave bonds of non-phenolic subunits of lignin only in the presence of a mediator [50,51]. Laccase redox mediators of natural origin are small molecular weight compounds which are lignin degradation products, plant phenolic secondary metabolites (e.g., vanillin, acetosyringone, and *p*-coumaric acid), or extracellular fungal metabolites, such as 4-hydroxybezylic alcohol, *p*-cinnamic acid, sinapic acid, syringaldehyde, or 3-hydroxyantharnillic acid [28,52]. These phenolic substances are oxidized by fungal laccase to phenoxyl radicals, which can oxidize non-phenolic residues of lignin, for example, through the hydrogen-abstraction mechanism [53]. Similar phenolic compounds are also observed within bacterial cultures capable of lignin degradation. For example, benzaldehyde with hydroxyl, methoxy, or trimethoxy substitutions is detected in *Aneurinibacillus aneurinilyticus, Pseudonomas putida*, *Bacillus* sp., *Streptomyces* sp., and *Paucimobilis* sp. cultures. The cinnamic acid with hydroxy and methoxy substitutions is reported in *Bacillus* sp. and *P. putida* cultures [54,55].

Some lignin-degradation-related phenolic compounds have mediation capacities when used in vitro but the presence of natural laccase mediators has not been proven during in vivo wood decay. When studying the sole action of laccase, it is difficult to exclude the possibility of interference from natural mediators, which are naturally present as phenolic compounds released from a lignin polymer [29].

## 3. Polyphenol oxidase (PPO) Properties and Physiological Functions

In the family of MCOs there are enzymes not only with laccase-like molecular structures but also those sharing similar catalytic functions. This group of enzymes, called polyphenol oxidases (PPOs), catalyzes the oxidation of phenols to quinones using molecular oxygen as a terminal electron acceptor [56]. PPOs are essential oxidases in biological systems, where they are involved in defense mechanisms, biosynthetic processes, polymerization, and detoxification of plant phenolic compounds [57]. In higher plants, the cross-linking of phenolic precursors is an important step in lignification [58]. PPOs are divided into three different groups: tyrosinases, catechol oxidases, and laccases, according to their substrate specificities and mechanisms of reactions [59,60]. Tyrosinases (E.C. 1.14.18.1; monophenol monooxygenase) catalyze the hydroxylation and oxidation of monophenols (including tyrosine, *p*-cresol, and *p*-coumaric acid) to *o*-diphenols and the oxidation of diphenols to the corresponding *o*-quinones [57]. Catechol oxidases (EC 1.10.3.1; 1,2-benzenediol: oxygen oxidoreductase also known as *o*-diphenol oxidase) solely catalyze the oxidation of *o*-diphenols to *o*-quinones (diphenolase activity). All PPO enzymes have an overlapping substrate spectrum and lack monophenol hydroxylating activity [61]. Cresolase and tyrosinases (EC 1.14.18.1) are the same enzymes differentiated according to their origin. Cresolases are ubiquitous PPOs in plants, whereas in animals, fungi, and bacteria, they are called tyrosinases [62]. Plant PPOs are predominantly located in the thylakoid membranes of chloroplasts, while the mammalian enzymes are usually present inside specialized melanocytes [63]. In some cases, polyphenol oxidases are secreted extracellularly [64].

PPOs oxidize phenolic or polyphenolic compounds, particularly flavonoids, which regulate all aspects of plant life [65]. These proteins are primarily responsible for enzymatic browning reactions and, therefore, play a leading role in plant defense against biotic and abiotic stresses [66]. During tissue injury, a melanin layer is built up as a protection against microbial pathogens [57]. Melanin polymers may contribute to the formation of protective barriers or be involved in the alkylation of proteins driven by PPOs, which in consequence may reduce the bioavailability of plant proteins for insects, or even the creation of a toxic environment for the invaders [67]. Oxidation of phenols to quinones activates a wound healing system and defense mechanisms in plants against herbivore insects and pathogens. Quinones bind covalently to leaf proteins and impede protein digestion in herbivores [68]. In addition to their role in the digestibility and palatability of plant tissues, melanin formation increases the cell wall resistance to insects and pathogens attack [69]. Later on, additional enzymes, such as phenoloxidase, quinone isomerase, and quinone methide isomerase, catalyze cross-linking reactions between quinonoid-reactive intermediates and cuticular components during sclerotization of insect cuticle in the wound healing process [70]. The same mechanism is observed when capsules are formed around parasites and parasitoids [71]. Quinones and other reactive intermediates (e.g., 5,6-dihydroxyindole) are more toxic to herbivores than the original phenolic substrates and may kill microbial pathogens and parasitoids directly [72]. Many PPOs cooperate with peroxidases (PODs; EC 1.11.1.7) [73] and have diverse and overlapping physiological functions in plants, which include involvement in redox metabolism, responses to wound healing, defense against pathogens or insects, synthesis of lignin and suberin, and cross-linking of cell wall components [74,75].

PPOs are the key enzymes in melanogenesis and represent heterogeneous polyphenolic polymers widely distributed in all living systems. In mammals, melanin is responsible for skin, eye, and hair pigmentation and has a fundamental role in the protection against UV radiation [76]. Melanin is also found in reptiles, amphibians, and fish. In insects, melanin is involved in the sclerotization of the cuticle, defense mechanisms, and wound healing [71]. Melanogenesis is related to the innate immunity and cell hemostasis in insects [77]. Both tyrosinase and laccase contribute to the formation of melanin pigments in fungal and bacterial cells [78], although melanogenesis is restricted to certain developmental stages of the mycelium, fruiting body formation, and wound healing [77]. Fungal melanin is quite abundant and appears in the cell wall rather than in specialized subcellular organelles such as animal melanosomes. Usually, melanin precursors are secreted and then oxidized outside the cell and melanin granules are deposited on the cell wall surface, where they are likely cross-linked with polysaccharides [77,79]. In fungi, melanin contributes to cell wall pigmentation and resistance against hydrolytic enzymes [43]. The same process in bacterial cells and spores plays an important protective role against environmental stress factors such as harmful UV radiation and ROS, and most likely protects against toxic heavy metals [61]. It has been suggested that extracellular polyphenol oxidases in bacteria participate in the polymerization and detoxification of plant phenolic compounds in soil environments [80].

It is believed that the catabolic role of fungal laccases consists of the degradation of natural polymers such as lignin, most likely in synergy with other lignolytic enzymes [81]. These enzymes are classified into two groups: heme peroxidases (PODs) and lignin-degrading auxiliary enzymes (LDAs). Heme peroxidases comprise lignin peroxidase (EC 1.11.1.14), manganese peroxidase (EC 1.11.1.13), versatile peroxidase (EC 1.11.1.16), and dye-decolorizing peroxidase (EC 1.11.1.19). In turn, the accessory enzymes implicated in lignin degradation include aryl-alcohol oxidase (EC 1.1.3.7), glyoxal oxidase (EC 1.2.3.5), pyranose 2-oxidase (EC 1.1.3.10), glucose dehydrogenase (EC 1.1.99.10), cellobiose dehydrogenase (EC 1.1.99.18), and heme-thiolate haloperoxidases [7]. All these oxidases reduce oxygen to H_2_O_2_ required by peroxidases, effectively coupling polysaccharide and lignin anabolism. Cellobiose dehydrogenases (EC 1.1.99.18) enhance this link by reduction of phenoxy radicals, cations of transition metals (e.g., FeIII, CuII, and MnIII), or quinones using electrons from the oxidation of cellobiose to cellobionolactone, a process that also contributes to the availability of redox mediators for laccases [82].

## 4. Bacterial Laccases

The occurrence of MCOs with laccase activity has also been described in prokaryotes [83,84]. Due to differences in the catalytic mechanism, these enzymes are often referred as “polyphenol oxidases”, “multicopper oxidases”, or “laccase-like enzymes”. However, they all catalyze oxidation of typical laccase substrates. The first prokaryotic protein with polyphenol oxidase activity (LMCO) was detected in non-motile *Azospirillum lipoferum* isolated from plant roots [85]. Isolated laccase occurs as a multimeric enzyme and its activity is correlated with production of a dark-brown pigment [85,86].

The well-known producers of prokaryotic laccase include gram-positive and gram-negative soil and aquatic bacteria belonging to the phyla α- and γ-proteobacteria, Firmicutes, Cyanobacteria, Aquificae, and Deinococcus-Thermus, as well as members of Archaea (Table S1). The presence of laccase is reported in the following species: *B. pumilus* [87,88], *B. subtilis* [89], *B. licheniformis* [90], *S. lavendulae* [91], *S. griseus* [92], *Escherichia coli* [83], *P. syringae* [93], *Thermus thermophilus* [94], *Sinorhizobium meliloti* [95], *Oscillatoria boryana* [96], *Haloferax volcanii* [97], and *Marinomonas mediterranea* [98], including species living in extreme habitats [94,99,100]. In prokaryotes, the cellular localization of laccase varies considerably among species (Table S1). It is probably related to the physiological role of the enzyme and appears to be dependent on the growth phase and the presence of inducing substrates. Most of the natively expressed laccases are present intracellularly as in *B. subtilis* [101], *S. meliloti* [95], *T. thermophilus* [94], and *M. mediterranea* [98]. Bacterial cells must have a strategy to cope with the intracellular presence of laccase due to its possible toxic byproducts. Rearrangement of the electron transport system has been suggested as a way in which the laccase-positive cells adapt to endogenous reactive quinones generated by laccases [102]. However, the extracellular localization of laccase is demonstrated in some bacilli and filamentous actinomycetes [103,104]. Laccase-like genes are identified in important human pathogens such as *E. coli*, *Bordetella pertusis*, *P. aeruginosa*, *Campylobacter jejuni*, *Yersinia pestis*, and *Mycobacterium leprae* [83]. The production of melanin and laccase activity, most likely, contributes to the virulence of these species [105].

The best known bacterial laccase is the CotA protein, which is localized at the outer coat of the endospore of *B. subtilis* [89] and other bacillus species. The 65 kDa CotA purified from an overproducing *E. coli* strain exhibits EPR spectra typical of the family of blue multicopper oxidases. The CotA structure has been elucidated with the use of comparative modeling. It contains all the features of a fungal laccase, including the surface-exposed copper center (T1) and two buried copper centers (T2 and T3) [101]. Analysis of the crystal structure revealed that the enzyme has a larger putative substrate-binding cavity than fungal or plant laccases [5]. Mutation of ligands in the T1 site impairs copper coordination, which alters the CotA biochemical properties drastically. The protein is highly thermostable with a half-life of about 2 h at 80 °C. The CotA recombinant laccase exhibits maximal activity for 2,2′-azino-bis(3-ethylbenzothiazoline-6-sulphonic acid (ABTS) and for syringaldazine (SGZ) oxidation at pH ≤3.0 and 7.0, respectively [101]. In contrast, SLAC protein has been identified in the genome of filamentous *S. coelicolor* [106]. This protein is a representative of the two-domain laccases with a substantially different protein architecture and appearing to be highly stable (Figure 1). The SLAC displays an unprecedentedly high pH optimum (9.4) for oxidation of 2,6-dimethoxyphenol (DMP); however, the recombinantly expressed enzyme exhibits paramagnetic properties typical for laccases [106]. Next to fungi, it is believed that actinomycetes are potent producers of laccases in nature [107], and their SLACs are thought to represent key evolutionary intermediates of the three-domain MCOs [16]. Still, their crystal structure resembles the structure of nitrite reductase or human ceruloplasmin more than that of a typical laccase [108,109].

The prokaryotic laccases described so far vary greatly in size from 32 to 180 kDa and occur as monomers, trimers, and tetramers. Although there is undeniable evidence for prokaryotic protein glycosylation [110], glycosylation of bacterial MCOs has not been investigated extensively and there are only few reports related to the carbohydrate content of bacterial laccases [97,107,111]. Similarly, very little is known about the electrochemistry of prokaryotic MCOs (Table S1). Bacterial (and plant) laccases belong to low-redox potential enzymes, with a redox potential at the T1 site (E^0′^_T1_) below 460 mV vs. NHE [108,112,113]. In comparison to fungal laccases, bacterial polyphenol oxidases are active at high pH values and much more stable at high temperatures. The high alkaline pH optimum (8.5 and 9 for ABTS and DMP, respectively) was shown for several *Streptomyces* laccases [106,107,114], whereas the highest temperature optimum (85 °C) was reported for McoP isolated form *Pyrobaculum aerophilum* [112] and *T. thermophilus* laccase (92 °C) [94]. Interestingly, non-melanogenic alkali-tolerant γ-proteobacterium JB isolated from soil drained with industrial wastewater synthesizes laccase stably at pH from 3 to 10.6 [100]. *S. ipomoea* laccase retains 100% activity in 1 M NaCl at pH 8.0 [115]. These extraordinary biochemical features underline prokaryotic laccases as a potential source of robust catalysts with possible biotechnological applications.

Bacterial MCOs with laccase activity are recognized as “moonlighting proteins” (i.e., multifunctional enzymes with multiple functions depending on their cellular localization) [116,117]. A probable mechanism of multifunctional enzyme switching has been hypothesized [116]. Although the possible functions of prokaryotic laccases were already discussed [13,84,118,119], their biological role in vivo remains obscure and speculative. Most of the bacterial proteins identified so far are not often directly linked with lignin degradation [120,121,122]. Although never proven, the role and efficacy of bacterial laccases in lignin degradation is nowadays heavily studied [13]. Modification of the lignin polymer in order to allow access of other enzymes to cellulose and hemicellulose was suggested recently [122]. In general, laccase corresponding gene products are mainly involved in metal homeostasis/oxidation, sporulation, morphogenesis, and cell and spore pigmentation and are linked to resistance to different stresses. The *Azospirillum* laccase was reported to be involved in cell pigmentation [85], utilization of naturally occurring plant phenolic compounds resulting from lignin metabolism [123], and/or electron transport [102]. These capabilities could be related to the competitiveness of *Azospirillum* sp. in the rhizosphere and may play an essential role in colonization of plant roots, especially when the oxygen concentration changes in the soil environment [86]. The role of *Streptomyces* laccase in morphogenesis and pigmentation [124], lignocellulose degradation [125], bacteria–bacteria interactions, or antibiotic production [126] was speculated as well. In turn, most of the laccases identified in *Bacillus* are a part of the spore’s outer coat that protects endospores from a diverse range of stresses. The *cotA* gene participates in the biosynthesis of a brown spore pigment, which is a melanin-like polymer responsible for protection against UV radiation. This view is supported by the direct observation of the protective effect of CotA in a laccase-positive strain of *B. subtilis*, which appears more resistant than *cotA*-deficient spores [89,101]. Moreover, brown-pigmented *Bacillus* sp. HR03 spores show remarkable resistance when exposed to hydrogen peroxide, UVA, and UVC. Therefore, the spore pigments in *Bacillus* are responsible for its resistance against harsh conditions, and laccase is an effective enzyme in the synthesis of these spore pigments [119,127]. The involvement of laccases in the cross-linking of spore coat proteins was postulated as well, which is somewhat analogous to the role of plant laccases in cell wall formation and is supported by the observation of tyrosine to di-tyrosine crosslinks in *B. subtilis* spores [12,128]. *B. subtilis* is capable of MnII oxidation, but its CotA is not involved in metal oxidation, unlike CumA, the spore coat protein of *Pseudomonas* sp. CumA is an MCO similar to laccase. It contributes to the oxidation of MnII, which may prolong the viability of the cell in the presence of ions of this metal [16,129]. Laccase-producing *B. halodurans* also show resistance against CuII toxicity [12]. Furthermore, laccase-like coding genes are also found in *E. coli* (PcoA and CueO, formerly YacK) and *P. syringae* (CopA). These pseudo-laccases structurally homologous to multicopper oxidases are important for bacterial copper resistance. A putative multicopper oxidase encoded by the *yacK* gene contains the predicted copper binding centers and displays phenol oxidase and ferroxidase activity. The enzyme moderates the sensitivity of *E. coli* to copper by exhibiting significant activity against siderophores utilized by *E. coli* for FeII uptake [130]. Moreover, *E. coli* CueO contains a Met and His-rich region, which partly covers the entrance to the T1 copper active site [131]. This region may provide additional Cu-binding sites and modify the active site structure upon CuII binding. *Pseudomonas* CopA shows limited but significant sequence homology with MCO proteins and is necessary for the expression of full copper resistance in these bacteria [132]. The role of laccase in cyanobacteria was investigated in relation to its bioremediation potential [133], suggesting its possible role in protecting the cell against harsh environmental conditions. However, only a few studies concerning cyanobacterial laccases have been reported [96,134].

## 5. Plant Laccase—Species Range and Roles

Laccase is isolated from gymnosperms and angiosperms, but in recent years, extracellular phenol oxidase from *Tetracystis aeria* (green algae) was confirmed as laccase according to the substrate specificity and properties of the purified enzyme [135]. Laccases have been detected in representatives of Anacardiaceae and other higher plants, including *Pinus taeda*, *Acer pseudoplatanus*, *Nicotiana tabacum*, *P. trichocarpa*, *Liridendron tulipifera*, *Lolium perenne*, *A. thaliana*, *Zea mays*, *Oryza sativa*, *Saccharum officinarum*, *Brassica napus*, and *Brachypodium distachyon* (Table S1) [136].

In 1883 Yoshida discovered that the first listed species in which laccase was detected was the Chinese lacquer tree *Rhus vernicifera*. Ten years later, Gabriel Bertrand isolated this enzyme from *R. succedanea* and other members of the Anacardiaceae family (e.g., *Mangifera indica*, *Schinus molle*, *Pistacia palaestina*, *Pleiogynium timoriense*) [137,138]. Laccase was located mostly in the resin ducts of these representatives. Production and secretion of laccase from cultures of *A. pseudoplatanus* was reported by Bligny and Douce [139] and the enzyme was later localized in xylem tissues of *P. taeda* [140], *P. euramericana* [141], and *N. tabaccum* [142]. The enzyme was also isolated from leaves of *Aesculus parviflora* [143].

The number of laccase isoforms in different plant species varies. For example, five laccases are expressed in the xylem of *P. trichocarpa* [141], eight laccases in the xylem tissues of *P. taeda* [32,140], and as many as 17 laccase genes in *A. thaliana* [144,145]. The molecular weight of plant laccases usually ranges between 60 and 130 kDa with an average composition of 500 to 600 aa [136]. The optimum pH value varies mostly between pH 5–7, and the isoelectric point (pI) ranges from 5 to 9.6; the enzymes are highly glycosylated (22%–45%) [136,137,145]. Plant laccases exhibit low redox potential of copper type I (T1) at about 430 mV vs. NHE. A characteristic feature of plant laccases is the ability to oxidize the substrate without the help of mediators [146].

**Figure 4:**
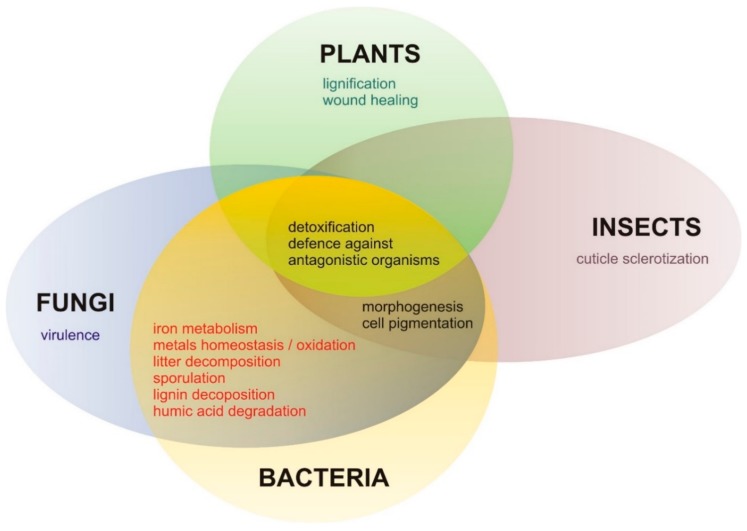
Comparison of laccase biological functions in different organisms. The protective role is common for all taxonomic groups and, most likely, it is the primary role of laccases in all living organisms. Other functions are most probably the result of specialization of this enzyme, which narrows its substrate spectrum.

Plant laccases are reported to be involved in (1) lignification responsible for maintenance of the cell wall structure and mechanical rigidity; (2) plant responses to environmental stresses and defense mechanisms; (3) wound healing; (4) iron metabolism; and (5) polymerization of phenolic compounds (Figure 4) [136,137,145,147,148,149]. The ability to oxidize lignin precursors suggests involvement of plant laccases in the lignification of the plant cell wall [32,140,141,150,151]. Laccase enzymes are secreted to the apoplast, where they catalyze the synthesis of lignin and regeneration of damaged plant tissues.

*A. thaliana* laccase is responsible for stem lignification while in *Populus* sp. it is present in other organs and tissues, such as the seed coat [145]. The presence of the enzyme in the resin ducts of Anacardiaceae indicates a defense function against herbivores, predators, and bacterial and fungal invasion [147]. It was found that during seed storage and germination, the *TT10* gene related to the formation of antimicrobial quinones participates in the creation of a barrier against pathogens in *Arabidopsis* [62]. In *A. thaliana*, laccases expressed in the seed-specific group are involved in the polymerization of seed coat flavonoids and the production of insoluble brown polymers with a putative protective function [144,145].

The main role of green algal laccases includes detoxification of phenolic compounds present in both terrestrial and aquatic environments, involvement in the synthesis of cell wall-associated polymers and UV-absorbing compounds, and metabolism of lignocellulosic substrates resulting in acquisition of nutrients. In algae, laccases may greatly contribute to the biotransformation of natural and xenobiotic aromatic compounds, including known environmental pollutants [135].

## 6. Fungal Laccases—Occurrence, Roles, Similarities, and Differences

Most of the information about fungal laccases originates from species belonging mainly to Basidiomycota and Ascomycota (Table S1). Fungal laccases are involved in the decomposition of lignocellulose polymers, defense/protection, virulence, pathogenesis, pigmentation, and sporulation processes (Figure 4). The main function of fungal laccase is biodegradation of lignocellulose and thus contribution to the carbon cycle in the biosphere [7,138,152,153]. The lignin polymer is highly resistant towards chemical and biological degradation, making wood decay a slow and biologically difficult process. Fungal laccases, especially those from white-rot species, are reported to exhibit high redox potential, close to 800 mV vs. NHE, facilitating abstraction of electrons from substrates, which may also act as redox mediators during the attack of laccase on lignin. As reported by Munk, Sitarz, Kalyani, Mikkelsen, and Meyer [29], fungal laccase can cleave bonds without the use of mediators in phenolic lignin model compounds. In the case of non-phenolic subunits of lignin, cleavage of covalent bonds by laccase is possible only by using mediators [29]. Moreover, laccase gene expression can be induced by natural plant derivatives (e.g., gallic or ferulic acids) that may occur in fungal cells during fungal infection [154].

Litter decomposition is the key step in nutrient recyclin (i.e., a highly complex process mediated by various fungal taxa with rapid succession of saprotrophic species) [155]. Among different organisms equipped with extracellular enzymes involved in this process, Ascomycota and Basidiomycota are dominant but also other fungi, especially Zygomycota and Glomeromycota, are observed [155,156,157,158]. Laccase genes were detected in litter-degrading fungi and the number of basidiomycete laccase genes was 5–10 times greater in organisms occurring in high-lignin forest floor than in a low-lignin environment [29,157]. Interestingly, aquatic fungal species were denied the ability to decompose lignin for many years. However, taxa belonging mainly to Ascomycota, Chytridiomycota, and Oomycota are major decomposers of litter and able to produce laccase as well [156]. Two laccase encoding gene fragments were found by Sole et al. [159] in pure cultures of *Clavariopsis aquatica* and it was suggested that laccase is a cell-associated enzyme [159,160]. Other fungi belonging to aquatic hyphomycetes [161] and aquatic Ascomycota, such as *Phoma* sp. and *Coniothyrium* [162], possess laccase activity as well.

In nature, the other important deposit of carbon is humic substances (HS), which are dark-colored organic materials formed during chemical and biological transformation of mainly plant residues but also animal and human wastes [163,164]. The main building blocks of HS are phenols, quinones, carbohydrates, as well as higher molecular mass compounds such as lignins, polysaccharides, melanins, and cutins. Microorganisms, especially fungi producing oxidizing enzymes, play the key role in HS formation, degradation, transformation, and finally mineralization [165]. Chefetz, Chen and Hadar [34] confirmed that laccase from *Chaetomium thermophilum* plays a significant role in the humification process by forming water-soluble polymers containing hydrophobic acids. In contrast, degradation and transformation of HS are catalyzed mainly by Basidiomycota enzymes. The pathway of transformation (humification vs. HS degradation) depends on substrate availability and such reaction conditions as the pH value, humidity, and presence of co-substrates [165]. Since pH plays a critical role, the engagement of different laccase isoenzymes may be important for improvement of the efficiency of degradation/transformation of HS [165]. Feng et al. [166] suggested a correlation between the abundance and diversity of fungal and bacterial laccase activity in arable subtropical soil, and the laccase activity was mainly of bacterial origin.

Laccase secretion is considered as one of the basic fungal responses to the presence of antagonistic conditions: other microorganisms, xenobiotics, metals, toxins, and biologically active compounds. Fungal laccases oxidize not only phenolic compounds but also non-phenolic substrates such as aromatic amines, polycyclic aromatic hydrocarbons, synthetic dyes, antibiotics, and other non-obvious laccase substrates [35,167]. In this way, laccase is a very useful enzymatic “tool” for elimination of natural or synthetic toxins occurring in the environment and is therefore involved in fungal active defense.

The induction of laccase secretion by cultures of *Agaricus bisporus, P. ostreatus*, and *Lentinula edodes* as a response to the presence of *Trichoderma* sp. was demonstrated [168,169,170]. As described by Sjaarda et al. [171], the expression of *A. bisporus* laccase genes is also induced by the presence of a toxic extract of *T. aggressivum* in the medium, and the resistance is correlated with the laccase activity. Interestingly, *T. viride* laccase secretion enhances in the presence of *Bacillus* sp. and *A. ochraceus* cells. Lakshmanan and Sadasivan [172] reported recently that inhibition of *T. viride* laccase causes the inability of this fungus to compete with antagonistic microorganisms.

*Botrytis cinerea* BcLCC2 laccase is an example of the involvement of this enzyme in defense against antibiotics such as 2,4-diacetylphloroglucinol (2,4-DAPG). The degradation of 2,4-DAPG occurs only in the presence of tannic acid used as the redox mediator [173]. Interestingly, laccase plays a role as a catalyst during cinnabarinic acid synthesis in *Pycnoporus cinnabarinus.* Cinnabarinic acid is an antibacterial compound, which may well protect this fungus from microbial predations [174,175]. Laccases are key enzymes in melanin metabolism. Many melanin pigments are antimicrobial and act as a virulence factor contributing also to the fungal species survival. They are found in bacterial endospores, fungal spores/conidia, or within cell walls and can secrete into the environment [176]. A correlation between *C. neoformans* melanisation and the ability to infect humans has been studied. This yeast-like fungus causes childhood infection and cryptococcal pneumonia-like disease and meningo-encephalitis (cryptococcosis) [177]. The expression of a single *CNLAC1* gene in a *C. neoformans* culture is induced by glucose starvation, acidification, and lowered temperature [178,179,180,181]. The pigment produced by *C. neoformans* in the presence of an exogenous substrate is characterized as a melanin-like compound, and laccase is identified as a main diphenol oxidase involved in its formation [182,183,184]. Notably, the melanin-like precursors include catecholamine, dopamine, adrenaline, and noradrenaline present in the mammalian central nervous system, which may explain the neurotropism of *C. neoformans* [180,185]. Laccase of *C. neoformans* also promotes virulence by reducing FeIII to FeII, thus inhibiting the oxidative burst in macrophages and, consequently, the enzyme has a negative effect on innate immunity [186]. *C. neoformans* laccase is tightly associated with the cell wall [187] and can therefore easily is part of the response to toxic hydroxyl radicals produced by macrophages [186].

Infection of AIDS patients caused by *Talaromyces marneffei* suggests a potential role of laccase in the virulence of this opportunistic pathogen [188,189,190]. A possible mechanism is associated with the production of a red soluble pigment by both yeast and mold forms of *T. marneffei*. To understand the role of laccase in virulence of *T. marneffei,* single, double, triple, and quadruple deletions of genes encoding lacA, lacB, lacC, and pbrB laccases were obtained. Only conidia from the quadruple mutant showed increased sensitivity to the antifungal agents and vulnerability to phagocytosis and killing by monocyte cell line THP-1 [188,190]. These results suggest that the laccase of *T. marneffei* promotes resistance of this pathogen to host immune defenses [188]. The pigment or other laccase products reduce immune recognition, potentially interfering with signaling pathways in monocytes [190]. Laccase genes are also found in the genome of *Fonsecaea* sp., which belongs to black yeast-like fungi with clinical importance [191].

*Colletotrichum gloeosporioides* is the main causal agent of anthracnose of *Mangifera indica* fruits [192]. Lac1 mutants with a disrupted *lac* gene (LAC1) are less pigmented, rarely produce conidia, and have reduced aerial mycelial mass and radial growth rates. The lac1 mutants are also less virulent during virulence tests on both wounded and non-wounded mango leaves and fruits. As reported by Kuo et al. [193], laccases in *Heterobasidion annosum* may act as a virulence factor during interactions with *P. sylvestris* seedlings [193]. Summarizing, inhibition of melanin synthesis in the fungal pathogen could be a viable approach to control diseases caused by fungi in plants and animals and may help to protect lumber against fungal decay.

Two phylogenetically closely related fungi, *T. atroviride* and *T. harzianum,* produce yellowish and green spores, respectively, but laccase activity is detected only during formation of darker green spores. The laccase activity is associated with the spore surface and is linked to the melanin-like polymer present in the cell wall or in the periplasm of the spore capsules. This research indicates that the spore-associated laccases are involved in the formation of the melanin pigment, which protects spores against temperature, UV light, and hydrolytic enzymes [194].

## 7. Occurrence and Function of Laccase in Animals

In the animal kingdom, insect laccases are most intensively studied. The enzyme is detected in members of the following insect genera: *Anophales, Apis*, *Bombyx*, *Calliphora*, *Diploptera*, *Drosophila*, *Lucilia*, *Menduca*, *Monochamus, Musca*, *Oryctes*, *Papilio*, *Phormia*, *Rhodnius*, *Sarcophaga*, *Schistocerca*, and *Tenebrio* [41,195,196,197,198,199,200,201]. They mostly belong to the orders *Hymenoptera*, *Diptera*, *Lepidoptera*, and *Coleoptera* (Table S1).

Several isoforms have also been described in mollusks [202], and the presence of the enzyme was confirmed in the lowest metazoan taxon (i.e., sponges). The laccase of *Suberites domuncula* showed high sequence similarity with the insect laccases, including *Bombus impatiens*, *Apis mellifera*, and *Acromyrmex echinatior*, and with nematode enzymes from *Caenorhabditis remanei*, *Ancylostoma ceylanicum*, and *Haemonchus contortus* [202]. Recently, genetic variation in the gene coding for human laccase has been associated with the risk of Crohn’s disease, leprosy, ulcerative colitis, and juvenile idiopathic arthritis [10]. In many mammalian species, putative sequences coding for this enzyme are identified (Table S1). Interestingly, laccase s found in epithelial cells of human and termite intestines [203,204]. In insect intestines, laccase plays a most likely protective role against toxic lignin derivatives resulting from a plant-based diet. Moreover, it is localized intracellularly [195,196] and the molecular mass identified in several insect species (including *M. sexta*) varies from 70 to 100 kDa [205]. The values of pH and pI vary between 5–6.5 and 5.1–6.3, respectively [49,198]. In comparison to plant and fungal laccases, insect enzymes have an extended amino-terminal region [198].

Sponges most likely utilize laccase as an antimicrobial agent [202] and for detoxification of xenobiotics and elimination of lignin-derived products from their filtered food. The best-described function of laccase in insects is its involvement in cuticle sclerotization in the epidermis of larval, pupal, and adult developmental stages of *Drosophila virilis* [27,136,198,201,206]. The sclerotization involves oxidative incorporation of acyldopamines: *N*-acetyldopamine (NADA, IV) and *N*-β-alanyldopamine into the cuticular matrix before ecdysis (pre-ecdysial sclerotization) or soon after ecdysis (post-ecdysial sclerotization). In some members of the order *Diptera*, sclerotization of soft larval cuticle occurs during puparium formation [207,208]. Laccase gene expression and activity in the *D. virilis, L. cuprina*, and *B. mori* life cycle is low in the intermolt period and increases drastically later during puparium formation to decrease again thereafter [198,207]. Two main forms of laccase are found in insects: laccase-1 and laccase-2. Laccase-2 is involved in cuticle tanning (e.g., in larval, pupal, and adult stages) of *T. castaneum* and *M. sexta* [41,198], while laccase-1 is expressed in the salivary glands, midgut, Malpighian tubules, fat body, and epidermis of *M. sexta*. Laccase-1 also oxidizes toxic compounds ingested by insects, thus playing a protective role in the insect gut [198,207,209]. Another well-known biological function of laccase in insects is production of melanin in the midgut as a primeval immune response against invasion of parasites [147], as described in *D. melanogaster* [210]. Studies of the laccase-2 gene from *M. sexta* and *B. mori* showed high expression in the epidermis prior to ecdysis. However, the cuticle of newly molted pupae does not have laccase activity, and the activity becomes detectable only several hours after ecdysis. These data suggest that cuticle laccase is synthesized as an inactive precursor, which is activated after the ecdysis stage [198,211]. In *Monochamus alternatus*, laccase-2 plays a role in pupal pigmentation and sclerotization of adult cuticle [199]. These two processes (sclerotization and pigmentation), known as cuticle tanning, involve the formation of covalent cross-links between polypeptides via oxidative and nucleophilic reactions of catechols and side-chain groups of amino acids. The protein conjugation causes hardening and darkening of the exoskeleton, cuticle, egg capsule, chorion, ootheca, and silk cocoon of insects [41]. Hattori et al. [212] identified a laccase in the salivary glands of *Nephotettix cincticeps*, which secretes a watery saliva involved in the detoxification of potentially toxic monolignols during the insect’s feeding [209]. A role of *M. sexta* laccase in the oxidation of toxic compounds in food and/or in iron metabolism has been proposed [151,198].

## 8. Conclusions

Scientific data suggest that laccase evolved as one of the first copper-containing enzymes in the oxygen era of the biosphere. With numerous organic and inorganic substrates, this enzyme is engaged in a variety of catalytic functions, which is generally described as protective against adverse environmental factors, including competitive or parasitic organisms and toxic compounds. The protective role of laccase has become so important that its’ genes have spread in numerous taxonomic groups, excluding only anaerobic organisms, in the course of evolution. Moreover, the low substrate specificity of laccases facilitates the use of this protein as an important virulence factor that protects infectious cells against host immune defense. Recent reports on laccase in humans may lead to the discovery of novel functions of this enzyme, which may be examined for the first time as part of the most complicated machinery of the human body.

Since numerous copies of laccase genes are present in many organisms, their further duplication in genomes is expected. This phenomenon may result in subfunctionalization of newly evolved laccases, which in consequence allow organisms to explore new food sources and protect themselves from future adverse conditions. Changes in the laccase structure, for example, duplication or deletion of cupredoxin-like domains, are possible, as it already happened in different taxa in the past.

## Supplementary Materials

Supplementary materials can be found at https://www.mdpi.com/1422-0067/21/3/966/s1, Table S1. Comparison of selected laccases from different taxa.

## Abbreviations

2,4-DAPG	2,4-diacetylphloroglucinol
ABTS	2,2’-azino-bis(3-ethylbenzothiazoline-6-sulphonic acid)
DMP	(2,6-dimethoxyphenol)
LDA	lignin-degrading auxiliary enzymes
LMCO	laccase-like multicopper oxidase
MCO	multicopper oxidase
NHE	normal hydrogen electrode
POD	ligninolytic class II peroxidases
PPO	polyphenol oxidase
ROS	reactive oxygen species
SGZ	syringaldazine
