# Supplementary material for: Laccase Properties, Physiological Functions, and Evolution
Source: Int J Mol Sci. 2020 Jan 31;21(3):966. doi: 10.3390/ijms21030966 (PMC7036934; doi:10.3390/ijms21030966)
Supplement: Supplementary file 1 [file ijms-21-00966-s001.pdf]

Table S1. Comparison of selected laccases from different taxa.

Origin and protein name	Cellular localization	Gene (number)	Aa length	MW (kDa)	pI	pH optimum	Temp. optimum (°C)	Glycosylation	Redox potential (mV vs. NHE)	References
**BACTERIA**<br>**Firmicutes**										
Bacillus subtilis, CotA	spore wall		511	65	7.7	7 (SGZ), ≤3 (ABTS)	75		455	[1-3]
Bacillus subtilis WD23	spore wall					6.8 (SGZ)	60			[4]
Bacillus subtilis MTCC 2414	extracellular			52-55		9 (guaiacol)	70			[5]
Bacillus subtilis SF	spore wall									[6]
Bacillus halodurans C-125 (recombinant)	intracellular		500	56		7.5–8.0 (SGZ)	45			[7]
Bacillus sphaericus	spore wall					6 (SGZ, DMP)	60			[8]
Bacillus licheniformis (recombinant)	intracellular			65	7.0	7 (SGZ, DMP), 4.2 (ABTS)				[9]
Bacillus pumilus, Cot A (recombinant)	intracellular	1	510	58		6.5 (SGZ), 4 (ABTS), 7 (DMP)	70			[10]
Bacillus tequilensis SN4, SN4LAC	extracellular			32		6.5 (SGZ), 5.5 (ABTS), 8 (DMP), 8 (guaiacol)	85			[11]
Paenibacillus glucanolyticus SLM1, CuOx (recombinant)		1	541	90		7 (ABTS)	40			[12]
**BACTERIA**<br>**Proteobacteria**<br>**α-proteobacteria**										

Sinorhizobium meliloti CE52G	intracellular/periplasmic			95		6 (SGZ), 5 (ABTS)				[13]
Sinorhizobium meliloti L3.8	periplasmic	1	649	70	4.77	2.2 (ABTS), 3.5 (caffeic acid), 5.0 (DMP), 5.5 (SGZ), and 6.0 (ferulic acid)	80	3.2%		[14]
Azospirillum lipoferum	intracellular			48.9 - 179.3		6 (SGZ)	30			[15,16]
**BACTERIA**<br>**Actinobacteria**<br>**Actinobacteria**										
Streptomyces lavendulae REN-7, STSL	mycelia-intracellular/intracellular			73		4.5 (catechol)	50			[17]
Streptomyces psammoticus	intracellular			43	7.9	8.5 (ABTS)	45	10%		[18]
Streptomyces coelicolor, SLAC	extracellular			69±8	6.2 - 8.2,	9.4 (DMP)			430	[19,20]
Streptomyces coelicolor (recombinant)	extracellular			32		9 (DMP), 4 (ABTS)	60			[21]
Streptomyces griseus, EpoA (recombinant)	cytoplasmic		348	114	5.3 - 5.6	6.5 (N,N-dimethyl-p-phenylenediamine sulfate)	40			[22]
Streptomyces ipomea, SilA	extracellular/cytoplasmic			77	6.18	7.5-8.0 (SGZ), 5 (ABTS), 8 (DMP)	60			[23]
Streptomyces cyaneus CECT3335	extracellular			75	5.6	4.5	70			[24]
Streptomyces sviceus, Ssl1 (recombinant)	intracellular	2		98.3		8 (SGZ), 9 (guaiacol,			375 ± 8	[25,26]

						DMP), 4 (ABTS),				
Corynebacterium glutamicum, CgL1 (recombinant)	intracellular	1	493	59			60			[27]
**BACTERIA**<br>**Proteobacteria**<br>**γ-proteobacteria**										
Marinomonas mediterranea MMB-1, PpoA (recombinant)	intracellular		675	54.3						[28,29]
γ-proteobacterium JB	intracellular			120		6 (SGZ)	55			[30]
Stenotrophomonas maltophilia AAP56	intracellular	1				7 (SGZ)	40			[31]
Escherichia coli W3110, CueO	periplasmic	1		53.4		6.5 (DMP)	55		440, 500	[32-34]
Escherichia coli, PcoA										[35]
Pseudomonas syringae, CopA			577	64						[33,36]
Pseudomonas putida GB-1, CumA		1								[37]
Pseudomonas putida F6	cytoplasmic			59		7 (SGZ)	30			[38]
Xanthomonas campestris, CopA		1	592	67						[36,39]
**BACTERIA**<br>**Aquificae**										
Aquifex aeolicus, McoA (recombinant)	intracellular (periplasmic-predicted)	1		57.8 (59.5 predicted)	4.2	7 (SGZ), 4 (ABTS)	75			[40]
**BACTERIA**<br>**Deinococcus-Thermus**										
Thermus thermophilus HB27	intracellular		462	53	7.18	5.5 (SGZ), 4.5 (ABTS)	92			[41]

**BACTERIA**<br>**Cyanobacteria**										
Arthrospira maxima SAE-25780	extracellular									[42]
Phormidium valderianum BDU 30501	intracellular									[43]
Oscillatoria boryana BDU92181	intracellular									[43]
Spirullina C11	intracellular									[44]
**ARCHAEA**<br>**Euryarchaeota**										
Haloferax volcanii, LccA (recombinant)	extracellular	1		75-80	acidic	6 (ABTS), 8.4 (SGZ)	45-50	6.9%		[45]
Pyrobaculum aerophilum, McoP (recombinant)	intracellular	1	477	49.6 (52.9 predicted)		7 (SGZ), 3 (ABTS)	85		398	[46]
**PLANTS**<br>**Algae**										
Tetracystis aeria	extracellular			220		2.5 (ABTS)<br>7.0 (SGZ)<br>6.5 (2,6-DMP)		A≥53%<br>B≥27%		[47]
**PLANTS**<br>**Higher plants**										
Acer pseudoplatanus	extracellular			97±5		6.6 (4-methylcatechol)		45%		[48]
	suspension cultures			115±15	5.2	5.2		40%		[49]

		3			4.5					[50]
Arabidopsis thaliana		17 Lcs *AtLac1-17* organ-specific *TT10*	553-586	64	7.0-9.6					[51,52]
Liriodendron tulipifera	xylem tissues	4	570-586	60.6-61.2	9.3-9.5					[53]
Pinus taeda	xylem			90	9	5.9		22%		[54]
	xylem	8	536-556	59.2-61.7	7.66-9.62					[55]
Populus canadensis	xylem									[50]
Populus euramericana		2		90 110	9.2	-		High manose type *N*-glycan		[50]
Populus trichocarpa	xylem	6								[56]
Prunus persica	fruit			73.5		6		25%		[57,58]
Rhus succedanea						6.4 (hydroquinone) 6.6 (catechol) 6.6 (L-ascorbate)				[59]
Rhus vernicifera				103-120	8.2-9.1			45-55		[60]
						7.5 (hydroquinone) 7.6 (catechol) 7.6 (L-ascorbate)				[59]
									T1: 394-434 T2: ~365 T3: 434-483	[61]

				110		7	25		415	[62]
Schinus molle				96-110		6.2				[63]
**FUNGI**<br>**Basidiomycota**										
Abortiporus biennis strain J2	extracellular	9		66		5	40			[64]
Agaricus blazei	extracellular			66	4.0	5.5	20			[65]
Armillaria mellea	extracellular	16		59	4.1	3.5				[66]
Athelia rolfsii<br>(= *Sclerotium rolfsii*), SRL	extracellular			55 -86	5.2	2.4 -4.5	62			[67]
Auricularia auricular-judae, Lcc	extracellular		575-801							[68]
Cerrena unicolor, Lac	extracellular	8	510-516	45-62	4.27-5.11	5.5	45-62	1.65-3.51		[69]
Gelatoporia subvermispora<br>(= *Ceriporiopsis subvermispora)*		8	518-525	71-79	3.4-4.82	3-6		10-15		[70-72]
Coprinus cinereus, CcLCC5		13	387-728	56.8		3	55			[73]
Coprinus friesii	extracellular			60	3.5	5-8				[74]
Cryptococcus neoformans	cell wall associated	4	594-641	75						[75]
Cyathus bulleri, lac	extracellular		473-584	5.0		3.5-5.5	30	16		[76]
Daedalea quercina	extracellular	4		69-71	3	2-7	60-70			[77]

Dichomitus squalens	extracellular	12	507-634	65-66	3.5-3.6	3		8-10.5		[78]
Flammulina velutipes, Flac	extracellular		496-670	28-45		5-6.5	30			[78]
Fomes fomentarius	extracellular	5				3.8-4.8	38-52			[79]
Fomitopsis pinicola	extracellular	6	539-604	92	3.8	3	80			[80]
Ganoderma lucidum, lac	extracellular		519-620	43-66		4.5-5.0	30-50			[81,82]
Gymnopus quercophilus<br>(= *Marasmius quercophilus*)			517	60-65		2.6-6.2	80			[83]
Heterobasidion annosum		22	513-560			4.5-5.3				[84]
Irpex lacteus	mycelium	1				3-7				[85]
Lentinula edodes, Lcc	extracellular	4	445-631	72.2	3	4	40	23.8		[86]
Lentinula edodes<br>(= *Lentinus edodes*), Lcc	intracellular	4	445-631	58-74	3.42	3-4.2	40-50	7.5-8.6		[87,88]
Lentinus sajor-caju<br>(= *Pleurotus sajor-caju*)	extracellular		465-532	55-61	4.7-5.61	5	40			[89,90]
Lentinus tigrinus<br>(= *Panus tigrinus*)	extracellular	7	394-568	63-69.1	3.15	3.75	55	6.9		[91,92]
Macrocybe gigantea<br>= *Tricholoma gigantea*			391	43		4	70			[93]
Merulius tremellosus<br>(= *Phlebia tremellosa*)			520	67	3.25					[94]
Panaeolus papilionaceus<br>*Panaeolus sphinctrinus*		2		60	<3.6<br><3.55	3-7<br>3-8				[74]

Perenniporia tephropora	extracellular			63	3.3	4-5				[95]
Phanerodontia chrysosporium (= *Phanerochaete chrysosporium*)	extracellular	5	444-624	66<br>46,5						[96]
Phlebia radiata	extracellular	5	520-548	60		5	70			[97]
Pleurotus eryngii	extracellular	10	523-533	61-65	4.1-4.2	3.5-4	55-65	1-7		[98]
Pleurotus floridanus (= *Pleurotus florida*)	extracellular		529-533	77-82	4.1	5	50			[99]
Pleurotus ostreatus, POXA	extracellular	13	514-533	40-67	4.1<br>4.3	3.6-8	25-50	10		[100-102]
Pleurotus pulmonarius,	extracellular		521-533	46		4-8	50	44		[103]
Pleurotus cornucopiae	fruit body			66		4	40			[104]
Pycnoporus sp.	extracellular					4-4.5	70			[105]
Pycnoporus cinnabarinus	extracellular	5	468-643	70-81	3.5-3.7	4-4.5	65	9		[106,107]
Pycnoporus coccineus	extracellular	6	511-518	61.8		4.5-5	65	10	723	[108]
Pycnoporus sanguineus	extracellular	5	518-570	59.5-68		2-5	65-71	7-9	729, 746	[108,109]
Rigidoporus lignosus, POX			515-521	51-60	3.1-3.7	6-7			700-730	[110]
Rhizoctonia solani,		17	416-623	50-100		6-7				[111]
Schizophyllum commune	extracellular and intracellular	2	518-583	62-64		5.4-6				[112]
Trametes troggi POXL	extracellular			53-70	3.3-4.5	2.5-3.5		12	760-790	[110,113,114]

Trametes hirsuta	extracellular		515-525	52-62		3-4.5	50-60			[115,116]
Trametes pubescens, LAP	extracellular	6	508-526	65-68	2.6	3-4.5	50	18		[117,118]
Trametes polyzona	extracellular	3	520	71		2-5				[119]
Trametes versicolor	extracellular	8	520-527	97-100	3.5-5.8	3-5.5	50			[120,121]
Trametes orientalis	extracellular			44		4	80			[122]
Trametes villosa	extracellular		473-520	63	3.5-6.5	5-5.5				[123]
Volvariella volvacea,		11	519	58	3.7	3-5.6	50			[124]
**FUNGI** **Ascomycota**										
Alternaria alternata		7	598-616	45		3.5-4.5	35-65			[125,126]
Aspergillus nidulans	conidia	4	594-609	36-80				12		[127,128]
Aspergillus flavus (= *Aspergillus oryzae*)	extracellular	6	556-620	60		3	50			[129]
Aspergillus ochraceus	intracellular			68		4	60			[130]
Aureobasidium pullulans		10	570-628	60-70		4.5	45-60			[131,132]
Botrytis cinerea	extracellular	11	561-581	35,5-38				70-80		[133]
Chaetomium thermophilum	extracellular			77-98	5.1	5-65	50-60			[134,135]
Cryphonectria parasitica	extracellular	15	567-591	77		2.5		24		[136]

Fusarium solani	extracellular	4	583-651	72		3	70	12		[137]
Gaeumannomyces graminis	extracellular	8	578-607	190	5.6	4,5				[138]
Melanocarpus albomyces			623	80	4	5-7	60-70			[139]
Neurospora crassa	extracellular	7	598-739	64-64.8				11		[140,141]
Ophiostoma novo-ulmi		3		70-79	5.1					[142]
Podospora anserina	mycelium	3	621	39-97.5		5.5- 7.5		23		[143,144]
Pyricularia grisea<br>(= *Magnaporthe grisea*)	extracellular	9		70		6	30			[145]
Trichoderma atroviride	extracellular	1-6				4.5				[146]
Trichoderma reesei	extracellular	4	589-623			4.5	25			[147]
Trichoderma harzianum	extracellular	1-4	653-720	79		4.5	35		692	[148]
Xylaria polymorpha	extracellular			67	3.1	2.5-5	55-60	7.5		[149]
**ANIMALS**<br>**Sponges**										
Suberites domuncula, LACC-1_SUBDO		1	785	87	4.8					[150]
**ANIMALS**<br>**Insects**										
Anophales gambiae, AgLac1		1	1009	110	5.52					[151]
Apis mellifera, Amlac2			731	81.5	6.05					[152]
Bemisia tabaci, LAC1	*salivary glands*		911	103		5 (ABTS)				[153]
Bombyx mori		2	763<br>740	70<br>84						[154]

Drosophila melanogaster	3rd instar larva (wandering) cuticle-bound suspension	1				6.5 (4-MC)				[155]
Manduca sexta						6.0				[156]
		2	801<br>760	88<br>81	5.15<br>6.36					[151]
	pupal cuticle purified recombinant	1		84		5 (NADA)<br>5.5 (NBAD)				[157]
Monochamus alternatus, MaLac2		1	741	81	6.12					[158]
Nephotettix cincticeps, NcLac	salivary glands (Sg)<br>midgut (Mg)<br>Malpighian tubules (Mt)<br>integument incl. cuticle and epidermis (Ep)	1	701-792	77-86	5.52-6.04					[159]
Tribolium castaneum, TcLac2A, TcLac2B		1		77		For TcLac2A<br>5.5 (Dopamine)<br>5.5 (NADA)<br>5.5 (NBAD)<br>7 (Dopa)<br>4.5 (ABTS)<br>For TcLac2B<br>6.0 (Dopamine)<br>5.5 (NADA)<br>5.5 (NBAD)<br>6.5 (Dopa)<br>4.5 (ABTS)			300 (Dopamine)<br>260 (NADA)<br>240 (NBAD)<br>670 (Dopa)	[160]

SUBSTRATES:

ABTS: 2,2´-azino-bis(3-ethylbenzothiazoline-6-sulphonic acid)

SGZ (syringaldazine): 4-hydroxy-3,5-dimethoxybenzaldehyde azine
2,6-DMP: 2,6-dimethoxyphenol
ACS: 3´,5´-dimethoxy-4´-hydroxyacetophenone (acetosyringone)
NADA: *N*-acetyldopamine
NBAD: *N*-β-alanyldopamine
4-MC: 4-methylcatechol

1. Martins, L.O.; Soares, C.M.; Pereira, M.M.; Teixeira, M.; Costa, T.; Jones, G.H.; Henriques, A.O. Molecular and biochemical characterization of a highly stable bacterial laccase that occurs as a structural component of the *Bacillus subtilis* endospore coat. *J. Biol. Chem.* **2002**, *277*, 18849-18859, doi:10.1074/jbc.M200827200.
2. Hullo, M.F.; Moszer, I.; Danchin, A.; Martin-Verstraete, I. CotA of *Bacillus subtilis* is a copper-dependent laccase. *Journal of bacteriology* **2001**, *183*, 5426-5430.
3. Melo, E.P.; Fernandes, A.T.; Durao, P.; Martins, L.O. Insight into stability of CotA laccase from the spore coat of *Bacillus subtilis*. *Biochem Soc T* **2007**, *35*, 1579-1582, doi:10.1042/Bst0351579.
4. Wang, C.; Zhao, M.; Li, D.; Cui, D.; Lu, L.; Wei, X. Isolation and characterization of a novel *Bacillus subtilis* WD23 exhibiting laccase activity from forest soil. *African Journal of Biotechnology* **2010**, *9*, 5496-5502.
5. Muthukumarasamy, N.P.; Jackson, B.; Joseph Raj, A.; Sevanan, M. Production of extracellular laccase from *Bacillus subtilis* MTCC 2414 using agroresidues as a potential substrate. *Biochemistry Research International* **2015**, *2015*, 9, doi:10.1155/2015/765190.
6. Held, C.; Kandelbauer, A.; Schroeder, M.; Cavaco-Paulo, A.; Guebitz, G.M. Biotransformation of phenolics with laccase containing bacterial spores. *Environmental Chemistry Letters* **2005**, *3*, 74-77, doi:10.1007/s10311-005-0006-1.
7. Ruijssenaars, H.J.; Hartmans, S. A cloned *Bacillus halodurans* multicopper oxidase exhibiting alkaline laccase activity. *Appl Microbiol Biot* **2004**, *65*, 177-182, doi:10.1007/s00253-004-1571-0.
8. Claus, H.; Filip, Z. The evidence of a laccase-like enzyme activity in a *Bacillus sphaericus* strain. *Microbiol Res* **1997**, *152*, 209-216.
9. Koschorreck, K.; Richter, S.M.; Ene, A.B.; Roduner, E.; Schmid, R.D.; Urlacher, V.B. Cloning and characterization of a new laccase from *Bacillus licheniformis* catalyzing dimerization of phenolic acids. *Appl Microbiol Biot* **2008**, *79*, 217-224, doi:10.1007/s00253-008-1417-2.
10. Reiss, R.; Ihssen, J.; Thöny-Meyer, L. Bacillus pumilus laccase: a heat stable enzyme with a wide substrate spectrum. *BMC Biotechnology* **2011**, *11*, 9, doi:10.1186/1472-6750-11-9.
11. Sondhi, S.; Sharma, P.; Saini, S.; Puri, N.; Gupta, N. Purification and characterization of an extracellular, thermo-alkali-stable, metal tolerant laccase from *Bacillus tequilensis* SN4. *Plos One* **2014**, *9*, e96951, doi:10.1371/journal.pone.0096951.
12. Mathews, S.L.; Smithson, C.E.; Grunden, A.M. Purification and characterization of a recombinant laccase-like multi-copper oxidase from *Paenibacillus glucanolyticus* SLM1. *J Appl Microbiol* **2016**, *121*, 1335-1345, doi:10.1111/jam.13241.
13. Castro-Sowinski, S.; Martinez-Drets, G.; Okon, Y. Laccase activity in melanin-producing strains of *Sinorhizobium meliloti*. *Fems Microbiol Lett* **2002**, *209*, 119-125, doi:10.1111/j.1574-6968.2002.tb11119.x.
14. Pawlik, A.; Wojcik, M.; Rulka, K.; Motyl-Gorzel, K.; Osinska-Jaroszuk, M.; Wielbo, J.; Marek-Kozaczuk, M.; Skorupska, A.; Rogalski, J.; Janusz, G. Purification and characterization of laccase from *Sinorhizobium meliloti* and analysis of the lacc gene. *Int J Biol Macromol* **2016**, *92*, 138-147, doi:10.1016/j.ijbiomac.2016.07.012.
15. Givaudan, A.; Effosse, A.; Faure, D.; Potier, P.; Bouillant, M.L.; Bally, R. Polyphenol oxidase in *Azospirillum lipoferum* isolated from rice rhizosphere - evidence for laccase activity in nonmotile strains of *Azospirillum lipoferum*. *Fems Microbiol Lett* **1993**, *108*, 205-210, doi:Doi 10.1016/0378-1097(93)90586-Q.
16. Diamantidis, G.; Effosse, A.; Potier, P.; Bally, R. Purification and characterization of the first bacterial laccase in the rhizospheric bacterium *Azospirillum lipoferum*. *Soil Biol Biochem* **2000**, *32*, 919-927, doi:Doi 10.1016/S0038-0717(99)00221-7.

17. Suzuki, T.; Endo, K.; Ito, M.; Tsujibo, H.; Miyamoto, K.; Inamori, Y. A thermostable laccase from *Streptomyces lavendulae* REN-7: purification, characterization, nucleotide sequence, and expression. *Bioscience, Biotechnology and Biochemistry* **2003**, *67*, 2167-2175.
18. Niladevi, K.; Sheejadevi, P.; Prema, P. Strategies for enhancing laccase yield from *Streptomyces psammoticus* and its role in mediator-based decolorization of azo dyes. *Applied Biochemistry and Biotechnology* **2008**, *151*, 9-19.
19. Machczynski, M.C.; Vijgenboom, E.; Samyn, B.; Canters, G.W. Characterization of SLAC: A small laccase from *Streptomyces coelicolor* with unprecedented activity. *Protein Sci* **2004**, *13*, 2388-2397, doi:10.1110/ps.04759104.
20. Gallaway, J.; Wheeldon, I.; Rincon, R.; Atanassov, P.; Banta, S.; Barton, S.C. Oxygen-reducing enzyme cathodes produced from SLAC, a small laccase from *Streptomyces coelicolor*. *Biosens Bioelectron* **2008**, *23*, 1229-1235, doi:10.1016/j.bios.2007.11.004.
21. Dubé, E.; Shareck, F.; Hurtubise, Y.; Daneault, C.; Beauregard, M. Homologous cloning, expression, and characterisation of a laccase from *Streptomyces coelicolor* and enzymatic decolourisation of an indigo dye. *Appl Microbiol Biot* **2008**, *79*, 597-603, doi:10.1007/s00253-008-1475-5.
22. Endo, K.; Hayashi, Y.; Hibi, T.; Hosono, K.; Beppu, T.; Ueda, K. Enzymological characterization of EpoA, a laccase-like phenol oxidase produced by *Streptomyces griseus*. *J Biochem* **2003**, *133*, 671-677, doi:10.1093/jb/mvg086.
23. Molina-Guijarro, J.M.; Perez, J.; Munoz-Dorado, J.; Guillen, F.; Moya, R.; Hernandez, M.; Arias, M.E. Detoxification of azo dyes by a novel pH-versatile, salt-resistant laccase from *Streptomyces ipomoea*. *International microbiology : the official journal of the Spanish Society for Microbiology* **2009**, *12*, 13-21, doi:10.2436/20.1501.01.77.
24. Arias, M.E.; Arenas, M.; Rodriguez, J.; Soliveri, J.; Ball, A.S.; Hernandez, M. Kraft pulp biobleaching and mediated oxidation of a nonphenolic substrate by laccase from *Streptomyces cyaneus* CECT 3335. *Applied and environmental microbiology* **2003**, *69*, 1953-1958.
25. Gunne, M.; Hoppner, A.; Hagedoorn, P.L.; Urlacher, V.B. Structural and redox properties of the small laccase Ssl1 from *Streptomyces sviceus*. *The FEBS journal* **2014**, *281*, 4307-4318, doi:10.1111/febs.12755.
26. Gunne, M.; Urlacher, V.B. Characterization of the alkaline laccase Ssl1 from *Streptomyces sviceus* with unusual properties discovered by genome mining. *Plos One* **2012**, *7*, e52360, doi:10.1371/journal.pone.0052360.
27. Ricklefs, E.; Winkler, N.; Koschorreck, K.; Urlacher, V.B. Expanding the laccase-toolbox: A laccase from *Corynebacterium glutamicum* with phenol coupling and cuprous oxidase activity. *Journal of biotechnology* **2014**, *191*, 46-53, doi:http://dx.doi.org/10.1016/j.jbiotec.2014.05.031.
28. Sanchez-Amat, A.; Solano, F. A pluripotent polyphenol oxidase from the melanogenic marine *Alteromonas* sp shares catalytic capabilities of tyrosinases and laccases. *Biochem. Biophys. Res. Commun.* **1997**, *240*, 787-792, doi:10.1006/bbrc.1997.7748.
29. Sanchez-Amat, A.; Lucas-Elio, P.; Fernandez, E.; Garcia-Borron, J.C.; Solano, F. Molecular cloning and functional characterization of a unique multipotent polyphenol oxidase from *Marinomonas mediterranea*. *Biochimica et biophysica acta* **2001**, *1547*, 104-116.
30. Bains, J.; Capalash, N.; Sharma, P. Laccase from a non-melanogenic, alkalotolerant gamma-proteobacterium JB isolated from industrial wastewater drained soil. *Biotechnol. Lett.* **2003**, *25*, 1155-1159.
31. Galai, S.; Limam, F.; Marzouki, M.N. A new *Stenotrophomonas maltophilia* strain producing laccase. Use in decolorization of synthetics dyes. *Applied Biochemistry and Biotechnology* **2009**, *158*, 416-431, doi:10.1007/s12010-008-8369-y.
32. Grass, G.; Rensing, C. CueO is a multi-copper oxidase that confers copper tolerance in *Escherichia coli*. *Biochemical and biophysical research communications* **2001**, *286*, 902-908, doi:DOI 10.1006/bbrc.2001.5474.
33. Alexandre, G.; Zhulin, I.B. Laccases are widespread in bacteria. *Trends in Biotechnology* **2000**, *18*, 41-42, doi:http://dx.doi.org/10.1016/S0167-7799(99)01406-7.

34. Miura, Y.; Tsujimura, S.; Kurose, S.; Kamitaka, Y.; Kataoka, K.; Sakurai, T.; Kano, K. Direct electrochemistry of CueO and its mutants at residues to and near type I Cu for oxygen-reducing biocathode. *Fuel Cells* **2009**, *9*, 70-78, doi:10.1002/fuce.200800027.
35. Huffman, D.L.; Huyett, J.; Outten, F.W.; Doan, P.E.; Finney, L.A.; Hoffman, B.M.; O'Halloran, T.V. Spectroscopy of Cu(II)-PcoC and the multicopper oxidase function of PcoA, two essential components of *Escherichia coli* pco copper resistance operon. *Biochemistry* **2002**, *41*, 10046-10055, doi:10.1021/bi0259960.
36. Solano, F.; Lucas-Elío, P.; López-Serrano, D.; Fernández, E.; Sanchez-Amat, A. Dimethoxyphenol oxidase activity of different microbial blue multicopper proteins. *Fems Microbiol Lett* **2001**, *204*, 175-181, doi:10.1111/j.1574-6968.2001.tb10882.x.
37. Brouwers, G.-J.; de Vrind, J.P.M.; Corstjens, P.L.A.M.; Cornelis, P.; Baysse, C.; de Vrind-de Jong, E.W. cumA, a gene encoding a multicopper oxidase, is involved in Mn(2+) oxidation in *Pseudomonas putida* GB-1. *Applied and environmental microbiology* **1999**, *65*, 1762-1768.
38. McMahon, A.M.; Doyle, E.M.; Brooks, S.; O'Connor, K.E. Biochemical characterisation of the coexisting tyrosinase and laccase in the soil bacterium *Pseudomonas putida* F6. *Enzyme and Microbial Technology* **2007**, *40*, 1435-1441.
39. Hsiao, Y.-M.; Liu, Y.-F.; Lee, P.-Y.; Hsu, P.-C.; Tseng, S.-Y.; Pan, Y.-C. Functional characterization of copA gene encoding multicopper oxidase in *Xanthomonas campestris* pv. *campestris*. *Journal of Agricultural and Food Chemistry* **2011**, *59*, 9290-9302, doi:10.1021/jf2024006.
40. Fernandes, A.T.; Soares, C.M.; Pereira, M.M.; Huber, R.; Grass, G.; Martins, L.O. A robust metallo-oxidase from the hyperthermophilic bacterium *Aquifex aeolicus*. *The FEBS journal* **2007**, *274*, 2683-2694, doi:10.1111/j.1742-4658.2007.05803.x.
41. Miyazaki, K. A hyperthermophilic laccase from *Thermus thermophilus* HB27. *Extremophiles* **2005**, *9*, 415-425, doi:10.1007/s00792-005-0458-z.
42. Afreen, S.; Anwer, R.; Singh, R.K.; Fatma, T. Extracellular laccase production and its optimization from *Arthrospira maxima* catalyzed decolorization of synthetic dyes. *Saudi Journal of Biological Sciences* **2018**, http://dx.doi.org/10.1016/j.sjbs.2016.01.015, doi:http://dx.doi.org/10.1016/j.sjbs.2016.01.015.
43. Palanisami, S.; Saha, S.K.; Lakshmanan, U. Laccase and polyphenol oxidase activities of marine cyanobacteria: a study with Poly R-478 decolourization. *World Journal of Microbiology and Biotechnology* **2010**, *26*, 63-69, doi:10.1007/s11274-009-0143-y.
44. Ansari, M.K.A.; Khatib, U.M.; Owens, G.; Fatma, T. Evaluation of methyl red tolerant cyanobacteria for simultaneous laccase production and dye decolorization. *International Journal of Waste Resources* **2016**, *6*, 2252-5211.
45. Uthandi, S.; Saad, B.; Humbard, M.A.; Maupin-Furlow, J.A. LccA, an archaeal laccase secreted as a highly stable glycoprotein into the extracellular medium by *Haloferax volcanii*. *Applied and environmental microbiology* **2010**, *76*, 733-743, doi:10.1128/AEM.01757-09.
46. Fernandes, A.T.; Damas, J.M.; Todorovic, S.; Huber, R.; Baratto, M.C.; Pogni, R.; Soares, C.M.; Martins, L.O. The multicopper oxidase from the archaeon *Pyrobaculum aerophilum* shows nitrous oxide reductase activity. *The FEBS journal* **2010**, *277*, 3176-3189, doi:10.1111/j.1742-4658.2010.07725.x.
47. Otto, B.; Schlosser, D. First laccase in green algae: purification and characterization of an extracellular phenol oxidase from *Tetracystis aeria*. *Planta* **2014**, *240*, 1225-1236, doi:10.1007/s00425-014-2144-9.
48. Bligny, R.; Douce, R. Excretion of laccase by sycamore (*Acer pseudoplatanus* L) cells .1. purification and properties of the enzyme. *Biochem J* **1983**, *209*, 489-496.
49. Sterjiades, R.; Dean, J.F.; Eriksson, K.E. Laccase from sycamore maple (*Acer pseudoplatanus*) polymerizes monolignols. *Plant physiology* **1992**, *99*, 1162-1168.

50. Ranocha, P.; McDougall, G.; Hawkins, S.; Sterjiades, R.; Borderies, G.; Stewart, D.; Cabanes-Macheteau, M.; Boudet, A.M.; Goffner, D. Biochemical characterization, molecular cloning and expression of laccases - a divergent gene family - in poplar. *European Journal of Biochemistry* **1999**, *259*, 485-495.
51. McCaig, B.C.; Meagher, R.B.; Dean, J.F. Gene structure and molecular analysis of the laccase-like multicopper oxidase (LMCO) gene family in *Arabidopsis thaliana*. *Planta* **2005**, *221*, 619-636, doi:10.1007/s00425-004-1472-6.
52. Berthet, S.; Thevenin, J.; Baratiny, D.; Demont-Caulet, N.; Debeaujon, I.; Bidzinski, P.; Leple, J.C.; Huis, R.; Hawkins, S.; Gomez, L.D., et al. Role of plant laccases in lignin polymerization. *Adv Bot Res* **2012**, *61*, 145-+, doi:10.1016/B8978-0-12-416023-1.00005-7.
53. LaFayette, P.R.; Eriksson, K.-E.L.; Dean, J.F.D. Characterization and heterologous expression of laccase cDNAs from xylem tissues of yellow-poplar (*Liriodendron tulipifera*). *Plant Molecular Biology* **1999**, *40*, 23-35, doi:10.1023/a:1026437406859.
54. Bao, W.; O'Malley D, M.; Whetten, R.; Sederoff, R.R. A laccase associated with lignification in loblolly pine xylem. *Science* **1993**, *260*, 672-674, doi:10.1126/science.260.5108.672.
55. Sato, Y.; Bao, W.L.; Sederoff, R.; Whetten, R. Molecular cloning and expression of eight laccase cDNAs in loblolly pine (*Pinus taeda*). *J Plant Res* **2001**, *114*, 147-155, doi:Doi 10.1007/Pl00013978.
56. Ranocha, P.; Chabannes, M.; Chamayou, S.; Danoun, S.; Jauneau, A.; Boudet, A.M.; Goffner, D. Laccase down-regulation causes alterations in phenolic metabolism and cell wall structure in poplar. *Plant physiology* **2002**, *129*, 145-155, doi:10.1104/pp.010988.
57. Mayer, A.M.; Harel, E. A laccase-like enzyme in peaches. *Phytochemistry* **1968**, *7*, 1253-&, doi:Doi 10.1016/S0031-9422(00)85620-4.
58. Lehman, E.; Harel, E.; Mayer, A.M. Copper content and other characteristics of purified peach laccase. *Phytochemistry* **1974**, *13*, 1713-1717, doi:Doi 10.1016/0031-9422(74)85078-8.
59. Omura, T. Studies on laccases of lacquer trees. I. Comparison of laccases obtained from *Rhus vernicifera* and *Rhus succedanea*. *J Biochem* **1961**, *50*, 264-272.
60. Yun-Yang, W.; Yu-Min, D.; Fang-Xing, Y.; Ying, X.; Rong-Zhi, C.; Kennedy, J.F. Purification and characterization of hydrosoluble components from the sap of Chinese lacquer tree *Rhus vernicifera*. *Int J Biol Macromol* **2006**, *38*, 232-240, doi:10.1016/j.ijbiomac.2006.02.019.
61. Reinhammar, B.R. Oxidation-reduction potentials of the electron acceptors in laccases and stellacyanin. *Biochimica et biophysica acta* **1972**, *275*, 245-259.
62. Nakamura, T. Purification and physico-chemical properties of laccase. *Biochim. Biophys. Acta* **1958**, *30*, 44-52.
63. Barnun, N.; Mayer, A.M.; Sharon, N. Properties of laccase in Schinus-Molle. *Phytochemistry* **1981**, *20*, 407-408, doi:Doi 10.1016/S0031-9422(00)84154-0.
64. Yin, L.; Ye, J.; Kuang, S.; Guan, Y.; You, R. Induction, purification, and characterization of a thermo and pH stable laccase from *Abortiporus biennis* J2 and its application on the clarification of litchi juice. *Bioscience, Biotechnology and Biochemistry* **2017**, *81*, 1033-1040, doi:10.1080/09168451.2017.1279850.
65. Ullrich, R.; Huong le, M.; Dung, N.L.; Hofrichter, M. Laccase from the medicinal mushroom *Agaricus blazei*: production, purification and characterization. *Appl Microbiol Biot* **2005**, *67*, 357-363, doi:10.1007/s00253-004-1861-6.
66. Rehman, A.U.; Thurston, C.F. Purification of laccase I from *Armillaria mellea*. *Journal of General Microbiology* **1992**, *138*, 1251-1257.
67. Ryan, S.; Schnitzhofer, W.; Tzanov, T.; Cavaco-Paulo, A.; Gübitz, G.M. An acid-stable laccase from *Sclerotium rolfsii* with potential for wool dye decolourization. *Enzyme and Microbial Technology* **2003**, *33*, 766-774, doi:10.1016/s0141-0229(03)00162-5.

68. Fan, X.; Zhou, Y.; Xiao, Y.; Xu, Z.; Bian, Y. Cloning, expression and phylogenetic analysis of a divergent laccase multigene family in *Auricularia auricula-judae*. *Microbiol Res* **2014**, *169*, 453-462, doi:10.1016/j.micres.2013.08.004.
69. Rogalski, J.; Janusz, G. Purification of extracellular laccase from *Cerrena unicolor*. *Preparative biochemistry & biotechnology* **2010**, *40*, 242-255, doi:10.1080/10826068.2010.488967.
70. Ruttimann-Johnson, C.; Salas, L.; Vicuna, R.; Kirk, T.K. Extracellular enzyme production and synthetic lignin mineralization by *Ceriporiopsis subvermispora*. *Applied and environmental microbiology* **1993**, *59*, 1792-1797.
71. Enoki, M.; Watanabe, T.; Nakagame, S.; Koller, K.; Messner, K.; Honda, Y.; Kuwahara, M. Extracellular lipid peroxidation of selective white-rot fungus, *Ceriporiopsis subvermispora*. *Fems Microbiol Lett* **1999**, *180*, 205-211.
72. Salas, C.; Lobos, S.; Larrain, J.; Salas, L.; Cullen, D.; Vicuna, R. Properties of laccase isoenzymes produced by the basidiomycete *Ceriporiopsis subvermispora*. *Biotechnology and applied biochemistry* **1995**, *21*, 323-333.
73. Lin, Y.; Zhang, Z.; Tian, Y.; Zhao, W.; Zhu, B.; Xu, Z.; Peng, R.; Yao, Q. Purification and characterization of a novel laccase from *Coprinus cinereus* and decolorization of different chemically dyes. *Molecular biology reports* **2013**, *40*, 1487-1494, doi:10.1007/s11033-012-2191-x.
74. Heinzkill, M.; Bech, L.; Halkier, T.; Schneider, P.; Anke, T. Characterization of laccases and peroxidases from wood-rotting fungi (family Coprinaceae). *Applied and environmental microbiology* **1998**, *64*, 1601-1606.
75. Zhu, X.; Gibbons, J.; Garcia-Rivera, J.; Casadevall, A.; Williamson, P.R. Laccase of *Cryptococcus neoformans* is a cell wall-associated virulence factor. *Infect. Immun.* **2001**, *69*, 5589-5596.
76. Salony; Mishra, S.; Bisaria, V.S. Production and characterization of laccase from *Cyathus bulleri* and its use in decolourization of recalcitrant textile dyes. *Appl Microbiol Biot* **2006**, *71*, 646-653, doi:10.1007/s00253-005-0206-4.
77. Baldrian, P. Purification and characterization of laccase from the white-rot fungus *Daedalea quercina* and decolorization of synthetic dyes by the enzyme. *Appl Microbiol Biot* **2004**, *63*, 560-563, doi:10.1007/s00253-003-1434-0.
78. Perie, F.H.; Reddy, G.V.; Blackburn, N.J.; Gold, M.H. Purification and characterization of laccases from the white-rot basidiomycete *Dichomitus squalens*. *Arch Biochem Biophys* **1998**, *353*, 349-355, doi:10.1006/abbi.1998.0625.
79. Rogalski, J.; Wojtas-Wasilewska, M.; Apalovič, R.; Leonowicz, A. Affinity chromatography as a rapid and convenient method for purification of fungal laccases. *Biotechnology and Bioengineering* **1991**, *37*, 770-777, doi:10.1002/bit.260370812.
80. Park, N.; Park, S.S. Purification and characterization of a novel laccase from *Fomitopsis pinicola* mycelia. *Int J Biol Macromol* **2014**, *70*, 583-589, doi:10.1016/j.ijbiomac.2014.06.019.
81. Fang, Z.; Liu, X.; Chen, L.; Shen, Y.; Zhang, X.; Fang, W.; Wang, X.; Bao, X.; Xiao, Y. Identification of a laccase Glac15 from *Ganoderma lucidum* 77002 and its application in bioethanol production. *Biotechnology for biofuels* **2015**, *8*, 54, doi:10.1186/s13068-015-0235-x.
82. Shrestha, P.; Joshi, B.; Joshi, J.; Malla, R.; Sreerama, L. Isolation and physicochemical characterization of laccase from *Ganoderma lucidum*-CDBT1 isolated from its native habitat in Nepal. *BioMed research international* **2016**, *2016*, 3238909, doi:10.1155/2016/3238909.
83. Farnet, A.-M.; Criquet, S.; Cigna, M.; Gil, G.; Ferré, E. Purification of a laccase from *Marasmius quercophilus* induced with ferulic acid: reactivity towards natural and xenobiotic aromatic compounds. *Enzyme and Microbial Technology* **2004**, *34*, 549-554, doi:10.1016/j.enzmictec.2003.11.021.
84. Johansson, M.; Denekamp, M.; Asiegbu, F.O. Production and isozyme pattern of extracellular laccase in the S and P intersterility groups of the root pathogen *Heterobasidion annosum*. *Mycological Research* **1999**, *103*, 365-371, doi:10.1017/s0953756298007436.

85. Svobodova, K.; Majcherczyk, A.; Novotny, C.; Kues, U. Implication of mycelium-associated laccase from Irpex lacteus in the decolorization of synthetic dyes. *Bioresource technology* **2008**, *99*, 463-471, doi:10.1016/j.biortech.2007.01.019.
86. Nagai, M.; Sato, T.; Watanabe, H.; Saito, K.; Kawata, M.; Enei, H. Purification and characterization of an extracellular laccase from the edible mushroom *Lentinula edodes*, and decolorization of chemically different dyes. *Appl Microbiol Biot* **2002**, *60*, 327-335, doi:10.1007/s00253-002-1109-2.
87. Nagai, M.; Kawata, M.; Watanabe, H.; Ogawa, M.; Saito, K.; Takesawa, T.; Kanda, K.; Sato, T. Important role of fungal intracellular laccase for melanin synthesis: purification and characterization of an intracellular laccase from *Lentinula edodes* fruit bodies. *Microbiology* **2003**, *149*, 2455-2462, doi:10.1099/mic.0.26414-0.
88. D'Annibale, A.; Celletti, D.; Felici, M.; Di Mattia, E.; Giovannozzi-Sermanni, G. Substrate specificity of laccase from *Lentinus edodes*. *Acta Biotechnologica* **1996**, *16*, 257-270, doi:10.1002/abio.370160408.
89. Fokina, O.; Eipper, J.; Kerzenmacher, S.; Fischer, R. Selective natural induction of laccases in *Pleurotus sajor-caju*, suitable for application at a biofuel cell cathode at neutral pH. *Bioresource technology* **2016**, *218*, 455-462, doi:10.1016/j.biortech.2016.06.126.
90. Murugesan, K.; Arulmani, M.; Nam, I.H.; Kim, Y.M.; Chang, Y.S.; Kalaichelvan, P.T. Purification and characterization of laccase produced by a white rot fungus *Pleurotus sajor-caju* under submerged culture condition and its potential in decolorization of azo dyes. *Appl Microbiol Biot* **2006**, *72*, 939-946, doi:10.1007/s00253-006-0403-9.
91. Quaratino, D.; Federici, F.; Petruccioli, M.; Fenice, M.; D'Annibale, A. Production, purification and partial characterisation of a novel laccase from the white-rot fungus *Panus tigrinus* CBS 577.79. *Antonie van Leeuwenhoek* **2007**, *91*, 57-69, doi:10.1007/s10482-006-9096-4.
92. Leontievsky, A.; Myasoedova, N.; Pozdnyakova, N.; Golovleva, L. `Yellow' laccase of *Panus tigrinus* oxidizes non-phenolic substrates without electron-transfer mediators. *Febs Lett* **1997**, *413*, 446-448, doi:10.1016/s0014-5793(97)00953-8.
93. Wang, H.X.; Ng, T.B. Purification of a novel low-molecular-mass laccase with HIV-1 reverse transcriptase inhibitory activity from the mushroom *Tricholoma giganteum*. *Biochemical and biophysical research communications* **2004**, *315*, 450-454, doi:10.1016/j.bbrc.2004.01.064.
94. Vares, T.; Niemenmaa, O.; Hatakka, A. Secretion of ligninolytic enzymes and mineralization of C-Ring-labelled synthetic lignin by three *Phlebia tremellosa* strains. *Applied and environmental microbiology* **1994**, *60*, 569-575.
95. Ben Younes, S.; Mechichi, T.; Sayadi, S. Purification and characterization of the laccase secreted by the white rot fungus *Perenniporia tephropora* and its role in the decolourization of synthetic dyes. *J Appl Microbiol* **2007**, *102*, 1033-1042, doi:10.1111/j.1365-2672.2006.03152.x.
96. Srinivasan, C.; Dsouza, T.M.; Boominathan, K.; Reddy, C.A. Demonstration of laccase in the white rot basidiomycete *Phanerochaete chrysosporium* BKM-F1767. *Applied and environmental microbiology* **1995**, *61*, 4274-4277.
97. Kaneko, S.; Cheng, M.; Murai, H.; Takenaka, S.; Murakami, S.; Aoki, K. Purification and characterization of an extracellular laccase from *Phlebia radiata* strain BP-11-2 that decolorizes fungal melanin. *Bioscience, Biotechnology and Biochemistry* **2009**, *73*, 939-942, doi:10.1271/bbb.80740.
98. Munoz, C.; Guillen, F.; Martinez, A.T.; Martinez, M.J. Laccase isoenzymes of *Pleurotus eryngii*: characterization, catalytic properties, and participation in activation of molecular oxygen and Mn^{2+} oxidation. *Applied and environmental microbiology* **1997**, *63*, 2166-2174.
99. Das, N.; Chakraborty, T.K.; Mukherjee, M. Purification and characterization of laccase-1 from *Pleurotus florida*. *Folia Microbiologica (Praha)* **2000**, *45*, 447-451.
100. Palmieri, G.; Cennamo, G.; Faraco, V.; Amoresano, A.; Sannia, G.; Giardina, P. Atypical laccase isoenzymes from copper supplemented *Pleurotus ostreatus* cultures. *Enzyme and Microbial Technology* **2003**, *33*, 220-230, doi:10.1016/s0141-0229(03)00117-0.

101. Liu, L.; Lin, Z.; Zheng, T.; Lin, L.; Zheng, C.; Lin, Z.; Wang, S.; Wang, Z. Fermentation optimization and characterization of the laccase from *Pleurotus ostreatus* strain 10969. *Enzyme and Microbial Technology* **2009**, *44*, 426-433, doi:10.1016/j.enzmictec.2009.02.008.
102. Pozdnyakova, N.N.; Turkovskaya, O.V.; Yudina, E.N.; Rodakiewicz-Nowak, Y. Yellow laccase from the fungus *Pleurotus ostreatus* D1: Purification and characterization. *Applied Biochemistry and Microbiology* **2006**, *42*, 56-61, doi:10.1134/s000368380601008x.
103. Marques De Souza, C.G.; Peralta, R.M. Purification and characterization of the main laccase produced by the white-rot fungus *Pleurotus pulmonarius* on wheat bran solid state medium. *Journal of basic microbiology* **2003**, *43*, 278-286, doi:10.1002/jobm.200390031.
104. Wong, J.H.; Ng, T.B.; Jiang, Y.; Liu, F.; Sze, S.C.; Zhang, K.Y. Purification and characterization of a Laccase with inhibitory activity toward HIV-1 reverse transcriptase and tumor cells from an edible mushroom (*Pleurotus cornucopiae*). *Protein and peptide letters* **2010**, *17*, 1040-1047.
105. Wang, Z.; Cai, Y.; Liao, X.; Zhang, F.; Zhang, D.; Li, Z. Production and characterization of a novel laccase with cold adaptation and high thermal stability from an isolated fungus. *Applied Biochemistry and Biotechnology* **2010**, *162*, 280-294, doi:10.1007/s12010-009-8801-y.
106. Sigoillot, C.; Record, E.; Belle, V.; Robert, J.L.; Levasseur, A.; Punt, P.J.; van den Hondel, C.A.; Fournel, A.; Sigoillot, J.C.; Asther, M. Natural and recombinant fungal laccases for paper pulp bleaching. *Appl Microbiol Biot* **2004**, *64*, 346-352, doi:10.1007/s00253-003-1468-3.
107. Eggert, C.; Temp, U.; Eriksson, K.E. The ligninolytic system of the white rot fungus *Pycnoporus cinnabarinus*: purification and characterization of the laccase. *Applied and environmental microbiology* **1996**, *62*, 1151-1158.
108. Uzan, E.; Nousiainen, P.; Balland, V.; Sipila, J.; Piumi, F.; Navarro, D.; Asther, M.; Record, E.; Lomascolo, A. High redox potential laccases from the ligninolytic fungi *Pycnoporus coccineus* and *Pycnoporus sanguineus* suitable for white biotechnology: from gene cloning to enzyme characterization and applications. *J Appl Microbiol* **2010**, *108*, 2199-2213, doi:10.1111/j.1365-2672.2009.04623.x.
109. Ramírez-Cavazos, L.I.; Junghanns, C.; Ornelas-Soto, N.; Cárdenas-Chávez, D.L.; Hernández-Luna, C.; Demarche, P.; Enaud, E.; García-Morales, R.; Agathos, S.N.; Parra, R. Purification and characterization of two thermostable laccases from *Pycnoporus sanguineus* and potential role in degradation of endocrine disrupting chemicals. *Journal of Molecular Catalysis B: Enzymatic* **2014**, *108*, 32-42, doi:10.1016/j.molcatb.2014.06.006.
110. Garzillo, A.M.; Colao, M.C.; Buonocore, V.; Oliva, R.; Falcigno, L.; Saviano, M.; Santoro, A.M.; Zappala, R.; Bonomo, R.P.; Bianco, C., et al. Structural and kinetic characterization of native laccases from *Pleurotus ostreatus*, *Rigidoporus lignosus*, and *Trametes trogii*. *Journal of protein chemistry* **2001**, *20*, 191-201.
111. Wahleithner, J.A.; Xu, F.; Brown, K.M.; Brown, S.H.; Golightly, E.J.; Halkier, T.; Kauppinen, S.; Pederson, A.; Schneider, P. The identification and characterization of four laccases from the plant pathogenic fungus *Rhizoctonia solani*. *Current genetics* **1996**, *29*, 395-403.
112. De Vries, O.M.H.; Kooistra, W.H.C.F.; Wessels, J.G.H. Formation of an extracellular laccase by a *Schizophyllum commune* dikaryon. *Microbiology* **1986**, *132*, 2817-2826, doi:10.1099/00221287-132-10-2817.
113. Garzillo, A.M.; Colao, M.C.; Caruso, C.; Caporale, C.; Celletti, D.; Buonocore, V. Laccase from the white-rot fungus *Trametes trogii*. *Appl Microbiol Biot* **1998**, *49*, 545-551.
114. Zouari-Mechichi, H.; Mechichi, T.; Dhouib, A.; Sayadi, S.; Martínez, A.T.; Martínez, M.J. Laccase purification and characterization from *Trametes trogii* isolated in Tunisia: decolorization of textile dyes by the purified enzyme. *Enzyme and Microbial Technology* **2006**, *39*, 141-148, doi:https://doi.org/10.1016/j.enzmictec.2005.11.027.
115. Schroeder, M.; Heumann, S.; Silva, C.J.; Cavaco-Paulo, A.; Guebitz, G.M. Specificities of a chemically modified laccase from *Trametes hirsuta* on soluble and cellulose-bound substrates. *Biotechnology letters* **2006**, *28*, 741-747, doi:10.1007/s10529-006-9052-4.

116. Liu, J.; Liu, W.; Cai, Y.; Liao, X.; Huang, Q.; Liang, X. Laccase production by *Trametes hirsuta*, characterization, and its capability of decoloring chlorophyll. *Polish journal of microbiology* **2014**, *63*, 323-333.
117. Si, J.; Peng, F.; Cui, B. Purification, biochemical characterization and dye decolorization capacity of an alkali-resistant and metal-tolerant laccase from *Trametes pubescens*. *Bioresource technology* **2013**, *128*, 49-57, doi:10.1016/j.biortech.2012.10.085.
118. Galhaup, C.; Goller, S.; Peterbauer, C.K.; Strauss, J.; Haltrich, D. Characterization of the major laccase isoenzyme from *Trametes pubescens* and regulation of its synthesis by metal ions. *Microbiology* **2002**, *148*, 2159-2169, doi:10.1099/00221287-148-7-2159.
119. Chairin, T.; Nitheranont, T.; Watanabe, A.; Asada, Y.; Khanongnuch, C.; Lumyong, S. Purification and characterization of the extracellular laccase produced by *Trametes polyzona* WR710-1 under solid-state fermentation. *Journal of basic microbiology* **2014**, *54*, 35-43, doi:10.1002/jobm.201200456.
120. Bertrand, B.; Martinez-Morales, F.; Tinoco, R.; Rojas-Trejo, S.; Serrano-Carreon, L.; Trejo-Hernandez, M.R. Induction of laccases in *Trametes versicolor* by aqueous wood extracts. *World Journal of Microbiology and Biotechnology* **2014**, *30*, 135-142, doi:10.1007/s11274-013-1420-3.
121. Han, M.J.; Choi, H.T.; Song, H.G. Purification and characterization of laccase from the white rot fungus *Trametes versicolor*. *Journal of Microbiology* **2005**, *43*, 555-560.
122. Zheng, F.; An, Q.; Meng, G.; Wu, X.J.; Dai, Y.C.; Si, J.; Cui, B.K. A novel laccase from white rot fungus *Trametes orientalis*: Purification, characterization, and application. *Int J Biol Macromol* **2017**, *102*, 758-770, doi:10.1016/j.ijbiomac.2017.04.089.
123. Yaver, D.S.; Xu, F.; Golightly, E.J.; Brown, K.M.; Brown, S.H.; Rey, M.W.; Schneider, P.; Halkier, T.; Mondorf, K.; Dalboge, H. Purification, characterization, molecular cloning, and expression of two laccase genes from the white rot basidiomycete *Trametes villosa*. *Applied and environmental microbiology* **1996**, *62*, 834-841.
124. Chen, S.; Ge, W.; Buswell, J.A. Biochemical and molecular characterization of a laccase from the edible straw mushroom, *Volvariella volvacea*. *European Journal of Biochemistry* **2004**, *271*, 318-328.
125. Diaz, L.; Del Rio, J.A.; Perez-Gilabert, M.; Ortuno, A. Involvement of an extracellular fungus laccase in the flavonoid metabolism in Citrus fruits inoculated with *Alternaria alternata*. *Plant Physiology and Biochemistry* **2015**, *89*, 11-17, doi:10.1016/j.plaphy.2015.02.006.
126. Tapwal, A.; Varghese, S.; Kumar, U.; Arora, J. Production of laccase by *Alternaria alternata* and *Lasiodiplodia theobromae*. *European Journal of Experimental Biology* **2014**, *4*, 196-201.
127. Thurston, C.F. The structure and function of fungal laccases. *Microbiology* **1994**, *140*, 19-26.
128. Kurtz, M.B.; Champe, S.P. Purification and characterization of the conidial laccase of *Aspergillus nidulans*. *Journal of bacteriology* **1982**, *151*, 1338-1345.
129. Gomaa, O.M.; Momtaz, O.A. Copper induction and differential expression of laccase in *Aspergillus flavus*. *Brazilian journal of microbiology : [publication of the Brazilian Society for Microbiology]* **2015**, *46*, 285-292, doi:10.1590/S1517-838246120120118.
130. Telke, A.A.; Kadam, A.A.; Jagtap, S.S.; Jadhav, J.P.; Govindwar, S.P. Biochemical characterization and potential for textile dye degradation of blue laccase from *Aspergillus ochraceus* NCIM-1146. *Biotechnology and Bioprocess Engineering* **2010**, *15*, 696-703, doi:10.1007/s12257-009-3126-9.
131. Ademakinwa, A.N.; Agboola, F.K. Biochemical characterization and kinetic studies on a purified yellow laccase from newly isolated *Aureobasidium pullulans* NAC8 obtained from soil containing decayed plant matter. *Journal, genetic engineering & biotechnology* **2016**, *14*, 143-151, doi:10.1016/j.jgeb.2016.05.004.

132. Rich, J.O.; Leathers, T.D.; Anderson, A.M.; Bischoff, K.M.; Manitchotpisit, P. Laccases from *Aureobasidium pullulans*. *Enzyme and Microbial Technology* **2013**, *53*, 33-37, doi:10.1016/j.enzmictec.2013.03.015.
133. Marbach, I.; Harel, E.; Mayer, A.M. Molecular properties of extracellular *Botrytis cinerea* laccase. *Phytochemistry* **1984**, *23*, 2713-2717, doi:https://doi.org/10.1016/0031-9422(84)83001-0.
134. Chefetz, B.; Chen, Y.; Hadar, Y. Purification and characterization of laccase from *Chaetomium thermophilium* and its role in humification. *Applied and environmental microbiology* **1998**, *64*, 3175-3179.
135. Ishigami, T.; Yamada, Y. Purification and properties of polyphenol oxidase from *Chaetomium thermophile*, a thermophilic fungus. *The Journal of General and Applied Microbiology* **1986**, *32*, 293-301, doi:10.2323/jgam.32.293.
136. Rigling, D.; Van Alfen, N.K. Extra- and intracellular laccases of the chestnut blight fungus, *Cryphonectria parasitica*. *Applied and environmental microbiology* **1993**, *59*, 3634-3639.
137. Wu, Y.R.; Luo, Z.H.; Kwok-Kei Chow, R.; Vrijmoed, L.L. Purification and characterization of an extracellular laccase from the anthracene-degrading fungus *Fusarium solani* MAS2. *Bioresource technology* **2010**, *101*, 9772-9777, doi:10.1016/j.biortech.2010.07.091.
138. Edens, W.A.; Goins, T.Q.; Dooley, D.; Henson, J.M. Purification and characterization of a secreted laccase of *Gaeumannomyces graminis* var. *tritici*. *Applied and environmental microbiology* **1999**, *65*, 3071-3074.
139. Kiiskinen, L.L.; Viikari, L.; Kruus, K. Purification and characterisation of a novel laccase from the ascomycete *Melanocarpus albomyces*. *Appl Microbiol Biot* **2002**, *59*, 198-204, doi:10.1007/s00253-002-1012-x.
140. Froehner, S.C.; Eriksson, K.E. Purification and properties of *Neurospora crassa* laccase. *Journal of bacteriology* **1974**, *120*, 458-465.
141. Lerch, K.; Deinum, J.; Reinhammar, B. The state of copper in *Neurospora laccase*. *Biochimica et biophysica acta* **1978**, *534*, 7-14.
142. Binz, T.; Canevascini, G. Purification and partial characterization of the extracellular laccase from *Ophiostoma novo-ulmi*. *Current Microbiology* **1997**, *35*, 278-281, doi:10.1007/s002849900254.
143. Minuth, W.; Klischies, M.; Esser, K. The phenoloxidases of the ascomycete *Podospora anserina*. Structural differences between laccases of high and low molecular weight. *European Journal of Biochemistry* **1978**, *90*, 73-82.
144. Molitoris, H.P.; Esser, K. [The phenoloxidases of the ascomycete *Podospora anserina*. V. Properties of laccase I after further purification]. *Archiv fur Mikrobiologie* **1970**, *72*, 267-296.
145. Iyer, G.; Chattoo, B.B. Purification and characterization of laccase from the rice blast fungus, *Magnaporthe grisea*. *Fems Microbiol Lett* **2003**, *227*, 121-126, doi:10.1016/s0378-1097(03)00658-x.
146. Holker, U.; Dohse, J.; Hofer, M. Extracellular laccases in ascomycetes *Trichoderma atroviride* and *Trichoderma harzianum*. *Folia Microbiologica (Praha)* **2002**, *47*, 423-427.
147. Ataci, N.; Sekme, S.; Arisan, I. Studies on laccase activity in the filamentous fungus *Trichoderma reesei*. *IUFS Journal of Biology* **2013**, *72*, 37-42.
148. Sadhasivam, S.; Savitha, S.; Swaminathan, K.; Lin, F.-H. Production, purification and characterization of mid-redox potential laccase from a newly isolated *Trichoderma harzianum* WL1. *Process Biochemistry* **2008**, *43*, 736-742, doi:10.1016/j.procbio.2008.02.017.
149. Liers, C.; Ullrich, R.; Pecyna, M.; Schlosser, D.; Hofrichter, M. Production, purification and partial enzymatic and molecular characterization of a laccase from the wood-rotting ascomycete *Xylaria polymorpha*. *Enzyme and Microbial Technology* **2007**, *41*, 785-793, doi:10.1016/j.enzmictec.2007.07.002.

150. Li, Q.; Wang, X.; Korzhev, M.; Schroder, H.C.; Link, T.; Tahir, M.N.; Diehl-Seifert, B.; Muller, W.E. Potential biological role of laccase from the sponge *Suberites domuncula* as an antibacterial defense component. *Biochim. Biophys. Acta* **2015**, *1850*, 118-128, doi:10.1016/j.bbagen.2014.10.007.
151. Dittmer, N.T.; Suderman, R.J.; Jiang, H.; Zhu, Y.C.; Gorman, M.J.; Kramer, K.J.; Kanost, M.R. Characterization of cDNAs encoding putative laccase-like multicopper oxidases and developmental expression in the tobacco hornworm, *Manduca sexta*, and the malaria mosquito, *Anopheles gambiae*. *Insect Biochem Molec* **2004**, *34*, 29-41.
152. Elias-Neto, M.; Soares, M.P.; Simoes, Z.L.; Hartfelder, K.; Bitondi, M.M. Developmental characterization, function and regulation of a Laccase2 encoding gene in the honey bee, *Apis mellifera* (Hymenoptera, Apinae). *Insect Biochem Molec* **2010**, *40*, 241-251, doi:10.1016/j.ibmb.2010.02.004.
153. Yang, C.H.; Guo, J.Y.; Chu, D.; Ding, T.B.; Wei, K.K.; Cheng, D.F.; Wan, F.H. Secretory laccase 1 in *Bemisia tabac*i MED is involved in whitefly-plant interaction. *Sci Rep-Uk* **2017**, *7*, 3623, doi:10.1038/s41598-017-03765-y.
154. Yatsu, J.; Asano, T. Cuticle laccase of the silkworm, *Bombyx mori*: purification, gene identification and presence of its inactive precursor in the cuticle. *Insect Biochem Molec* **2009**, *39*, 254-262, doi:10.1016/j.ibmb.2008.12.005.
155. Sugumaran, M.; Giglio, L.; Kundzicz, H.; Saul, S.; Semensi, V. Studies on the enzymes involved in puparial cuticle sclerotization in *Drosophila melanogaster*. *Archives of insect biochemistry and physiology* **1992**, *19*, 271-283, doi:10.1002/arch.940190406.
156. Thomas, B.R.; Yonekura, M.; Morgan, T.D.; Czapla, T.H.; Hopkins, T.L.; Kramer, K.J. A trypsin-solubilized laccase from pharate pupal integument of the tobacco hornworm, *Manduca sexta*. *Insect Biochem* **1989**, *19*, 611-&, doi:Doi 10.1016/0020-1790(89)90095-4.
157. Dittmer, N.T.; Gorman, M.J.; Kanost, M.R. Characterization of endogenous and recombinant forms of laccase-2, a multicopper oxidase from the tobacco hornworm, *Manduca sexta*. *Insect Biochem Molec* **2009**, *39*, 596-606, doi:10.1016/j.ibmb.2009.06.006.
158. Niu, B.L.; Shen, W.F.; Liu, Y.; Weng, H.B.; He, L.H.; Mu, J.J.; Wu, Z.L.; Jiang, P.; Tao, Y.Z.; Meng, Z.Q. Cloning and RNAi-mediated functional characterization of MaLac2 of the pine sawyer, *Monochamus alternatus*. *Insect Molecular Biology* **2008**, *17*, 303-312, doi:10.1111/j.1365-2583.2008.00803.x.
159. Hattori, M.; Tsuchihara, K.; Noda, H.; Konishi, H.; Tamura, Y.; Shinoda, T.; Nakamura, M.; Hasegawa, T. Molecular characterization and expression of laccase genes in the salivary glands of the green rice leafhopper, *Nephotettix cincticeps* (Hemiptera: Cicadellidae). *Insect Biochem Molec* **2010**, *40*, 331-338, doi:10.1016/j.ibmb.2010.02.009.
160. Gorman, M.J.; Sullivan, L.I.; Nguyen, T.D.; Dai, H.; Arakane, Y.; Dittmer, N.T.; Syed, L.U.; Li, J.; Hua, D.H.; Kanost, M.R. Kinetic properties of alternatively spliced isoforms of laccase-2 from *Tribolium castaneum* and *Anopheles gambiae*. *Insect Biochem Molec* **2012**, *42*, 193-202, doi:10.1016/j.ibmb.2011.11.010.
